# *Para*-cresol production by *Clostridium difficile* affects microbial diversity and membrane integrity of Gram-negative bacteria

**DOI:** 10.1371/journal.ppat.1007191

**Published:** 2018-09-12

**Authors:** Ian J. Passmore, Marine P. M. Letertre, Mark D. Preston, Irene Bianconi, Mark A. Harrison, Fauzy Nasher, Harparkash Kaur, Huynh A. Hong, Simon D. Baines, Simon M. Cutting, Jonathan R. Swann, Brendan W. Wren, Lisa F. Dawson

**Affiliations:** 1 Department of Pathogen Molecular Biology, London School of Hygiene and Tropical Medicine, London, United Kingdom; 2 Department of Surgery & Cancer, Imperial College London, London, United Kingdom; 3 Bioinformatics and Next Generation sequencing core facility, National Institute for Biological Standards and Control South Mimms, Potters Bar, United Kingdom; 4 Department of Biomedical Sciences, Royal Holloway University of London, Egham, United Kingdom; 5 Department of Biological and Environmental Sciences, University of Hertfordshire, Hatfield, United Kingdom; University of Texas Medical School at Houston, UNITED STATES

## Abstract

*Clostridium difficile* is a Gram-positive spore-forming anaerobe and a major cause of antibiotic-associated diarrhoea. Disruption of the commensal microbiota, such as through treatment with broad-spectrum antibiotics, is a critical precursor for colonisation by *C*. *difficile* and subsequent disease. Furthermore, failure of the gut microbiota to recover colonisation resistance can result in recurrence of infection. An unusual characteristic of *C*. *difficile* among gut bacteria is its ability to produce the bacteriostatic compound *para*-cresol (*p*-cresol) through fermentation of tyrosine. Here, we demonstrate that the ability of *C*. *difficile* to produce *p*-cresol *in vitro* provides a competitive advantage over gut bacteria including *Escherichia coli*, *Klebsiella oxytoca* and *Bacteroides thetaiotaomicron*. Metabolic profiling of competitive co-cultures revealed that acetate, alanine, butyrate, isobutyrate, *p-*cresol and *p*-hydroxyphenylacetate were the main metabolites responsible for differentiating the parent strain *C*. *difficile* (630Δ*erm*) from a defined mutant deficient in *p-*cresol production. Moreover, we show that the *p*-cresol mutant displays a fitness defect in a mouse relapse model of *C*. *difficile* infection (CDI). Analysis of the microbiome from this mouse model of CDI demonstrates that colonisation by the *p-*cresol mutant results in a distinctly altered intestinal microbiota, and metabolic profile, with a greater representation of Gammaproteobacteria, including the Pseudomonales and Enterobacteriales. We demonstrate that Gammaproteobacteria are susceptible to exogenous *p*-cresol *in vitro* and that there is a clear divide between bacterial Phyla and their susceptibility to *p*-cresol. In general, Gram-negative species were relatively sensitive to *p*-cresol, whereas Gram-positive species were more tolerant. This study demonstrates that production of *p*-cresol by *C*. *difficile* has an effect on the viability of intestinal bacteria as well as the major metabolites produced *in vitro*. These observations are upheld in a mouse model of CDI, in which *p*-cresol production affects the biodiversity of gut microbiota and faecal metabolite profiles, suggesting that *p*-cresol production contributes to *C*. *difficile* survival and pathogenesis.

## Author summary

*Clostridium difficile* is a bacterium responsible for causing the majority of antibiotic associated diarrhoea outbreaks world-wide. In the United States of America, *C*. *difficile* infects half a million people annually. Antibiotics disrupt the natural protective gut microbiota, rendering people susceptible to *C*. *difficile* infection, which leads to potentially life-threatening disease and complications. *C*. *difficile* is transmitted by spores, which are able to survive in harsh environments for long periods of time. After initial treatment for *C*. *difficile*, up to 35% of patients develop the disease again, thus requiring additional and more successful treatment. Here, we use novel techniques to show that *C*. *difficile* produces a compound, *p*-cresol, which has detrimental effects on the natural protective gut bacteria. We show that *p*-cresol selectively targets certain bacteria in the gut and disrupts their ability to grow. By removing the ability of *C*. *difficile* to produce *p*-cresol, we show that it makes *C*. *difficile* less able to recolonise after an initial infection. This is linked to significant alterations in the natural healthy bacterial composition of the gut. Our study provides new insights into the effects of *p*-cresol production on the healthy gut microbiota and how it contributes to *C*. *difficile* survival and pathogenesis.

## Introduction

*Clostridium difficile* is a Gram-positive spore-forming enteric pathogen and the leading cause of antibiotic-associated diarrhoea worldwide[1]. *C*. *difficile* infection (CDI) ranges from self-limiting diarrhoea to severe and life threatening pseudomembranous colitis[2]. *C*. *difficile* spores are the aetiological agent of CDI transmission and are resistant to desiccation, environmental stress, disinfectants and heat[3, 4]. These spores, present in both hospitals and the environment are transmitted via the faecal-oral route, contributing to both nosocomial and community acquired CDI [3]. Infection with *C*. *difficile* is frequently preceded by treatment with broad-spectrum antibiotics, which eliminate discrete taxa of the commensal intestinal microbiota resulting in dysbiosis and permitting colonisation by *C*. *difficile*. Certain bacterial taxa have been highlighted as important in the prevention of *C*. *difficile* colonisation[5–7]. Since restoration of microbial diversity can resolve recurrent infections, faecal transplantation is viewed as an effective treatment strategy[8]. However, a greater understanding of how *C*. *difficile* is able to influence the gut microbiota and disrupt intestinal homeostasis is a current imperative.

Human intestinal bacteria have been shown to ferment dietary-derived carbohydrates[9] and proteins[10], producing short chain fatty acids (SCFA), as well as an array of metabolites via fermentation of aromatic amino acids[11]. The secondary metabolites of this highly diverse microbial community have the potential to either positively or negatively influence many aspects of human health [12], with some demonstrated to possess toxic and carcinogenic properties [11, 13, 14]. Aromatic amino acids such as phenylalanine, tryptophan and tyrosine are important sources of phenyl metabolites. These metabolites can be absorbed in the small intestine or pass through to the colon[15] to be excreted in faeces. One such fermentation product, phenylacetic acid (PAA), is the most commonly detected secondary metabolite in healthy human faeces, with reported concentrations of 479 μM[15]. *C*. *difficile* ferments tyrosine, via *p*-hydroxyphenylacetate (*p*-HPA), to produce *p*-cresol. *Para*-cresol is a phenolic compound [16] that has been demonstrated to inhibit the growth of a range of bacterial species and other microorganisms[17, 18]. To date, the capacity to produce *p-*cresol has only been demonstrated in a select number of organisms[19, 20], including eighteen intestinal commensal species[11]. However, the *in vitro* production of *p*-cresol by these species was relatively low (ranging from 0.06–1.95 μg/ml)[11]. Furthermore, *C*. *difficile* can tolerate relatively high concentrations (1 mg/ml) of *p*-cresol[21, 22]. As such, the ability to synthesise and tolerate high concentrations of *p*-cresol has led to the hypothesis that it may provide *C*. *difficile* with a competitive advantage over other microorganisms.

The enzyme responsible for the decarboxylation of *p*-HPA is a member of the glycyl radical family, 4-hydroxyphenylacetate decarboxylase, which is encoded by three genes *hpdB* (*CD630_01530*), *hpdC* (*CD630_01540*) *and hpdA* (*CD630_01550*), which are co-transcribed in an operon. The *hpdBCA* operon is highly conserved in all the sequenced *C*. *difficile* isolates. We have previously shown that disruption of any of the three genes renders *C*. *difficile* incapable of synthesising *p*-cresol[22]. In this study, we demonstrate that production of *p-*cresol by *C*. *difficile* confers a fitness advantage over other intestinal bacteria both *in vitro* and *in vivo*, specifically those with a Gram-negative cell envelope. The treatment of human faecal samples with exogenous *p-*cresol significantly modified the cultivable bacteria therein, in a species-specific manner. Furthermore, a *p*-cresol deficient mutant showed a modest but significant reduction in viable counts in a relapse mouse model of CDI. Comparisons of the metabolic and 16S rRNA profiles identified variation in the biochemical and bacterial composition between mice infected with the *C*. *difficile* strain 630Δ*erm* and the *p-*cresol deficient mutant (*hpdC*::CT) following infection and relapse. This is the first study to show that *p-*cresol production is a mechanism by which *C*. *difficile* confers a competitive advantage over other gut bacteria.

## Results

### Exogenous *p*-cresol inhibits the growth of Gram-negative intestinal commensal bacteria

**Fig 1:**
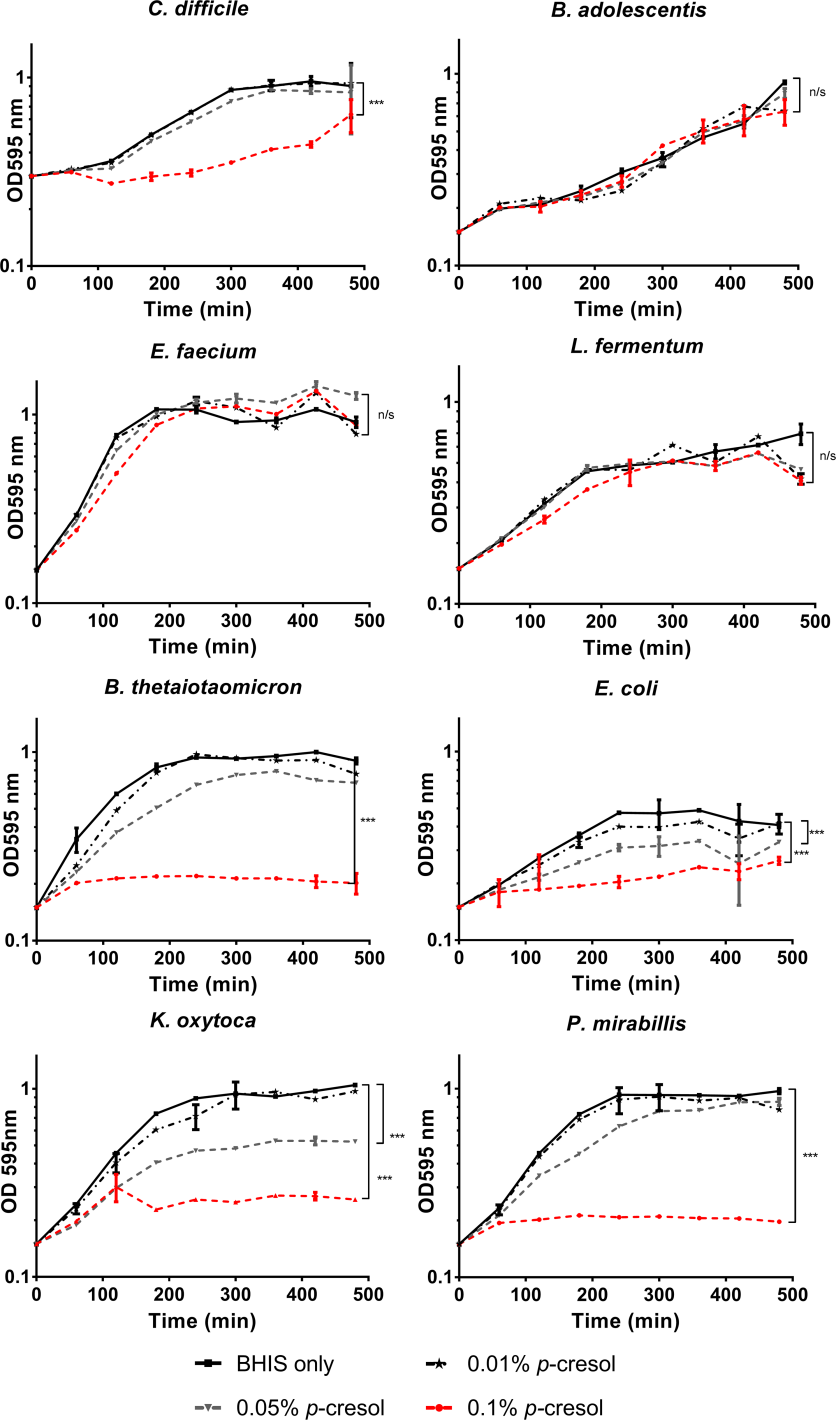
Growth of gut commensals in medium supplemented with exogenous *p*-cresol. Growth of gut commensals in BHIS compared to media containing 0.01%, 0.5% and 0.1% (v/v) *p-*cresol. Each curve represents the mean growth rate of three independent replicates. Regression analysis was used to determine significant differences in growth rate compared to the BHIS control over the course of the experiment and marked *** *p*<0.01. Error bars represent the standard deviation.

It has been hypothesised that *p*-cresol production provides *C*. *difficile* with a selective advantage over competitors in the human gut. To investigate this, we assessed the effect of exogenous *p*-cresol on the *in vitro* growth dynamics of selected intestinal commensal species (S1 Table) compared to *C*. *difficile* strain 630Δ*erm* (Fig 1 & S2 Table). The data shows a clear pattern whereby sensitivity to *p*-cresol correlated with bacterial cell envelope structure. We observed that Gram-positive bacteria were significantly more tolerant to *p*-cresol than Gram-negative bacteria (Coefficient of variance (COV) = 0.599, *p<0*.*001*). Growth of the Gram-negative species, including members of the Bacteroidaceae (*Bacteroides thetaiotaomicron*) and Enterobacteriaceae (*Escherichia coli*, *Klebsiella oxytoca* and *Proteus mirabilis*) families were inhibited by the addition of exogenous *p*-cresol in a dose-dependent manner (Fig 1 & S3 Table) and demonstrated a significant decrease in cell growth compared to *C*. *difficile* (*p*<0.005). In contrast, the Gram-positive species including those from the Bifidobacteriaceae (*Bifidobacterium adolescentis*), Enterococcaceae (*Enterococcus faecium*) and Lactobacillaceae (*Lactococcus fermentum*) families displayed no significant reduction in growth rate, even at 0.1% (v/v) *p*-cresol (Fig 1). Interestingly, *E*. *faecium* displayed greater tolerance to *p-*cresol than *C*. *difficile* itself (COV = 0.6 *p* = 0.002, S3 Table).

**Fig 2:**
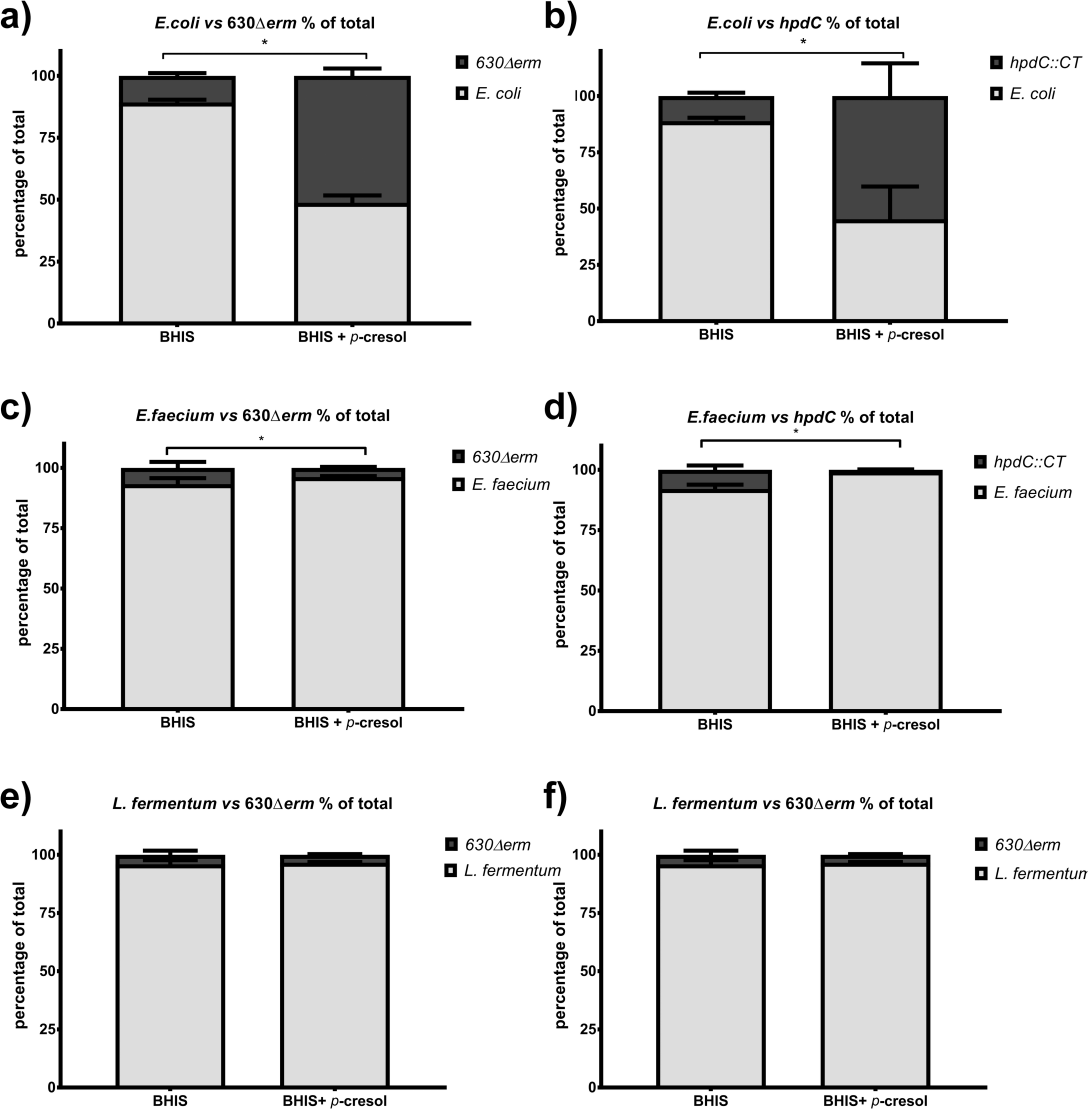
Co-culture assays in medium supplemented with exogenous *p*-cresol. Relative fitness of *C*. *difficile* 630Δ*erm* and *hpdC*::CT when grown in co-culture with gut commensals, with and without 0.05% (v/v) *p-*cresol. The relative fitness of 630Δ*erm* and *hpdC*::CT grown in co-culture with a&b) *E*. *coli*, c&d) *E*. *faecium*, e&f) *L*. *fermentum* (e&f). Viable counts are represented by CFU/ml and displayed as a percentage of the total culture. Error bars are representative of three independent replicates. Regression analysis was used to determine significant differences in growth taking strain and media into consideration ***p<0.001.

We had previously constructed a ClosTron inactivation mutant in the *hpd*C decarboxylase gene (strain *hpdC*::CT), which renders *C*. *difficile* unable to produce *p*-cresol[22]. To investigate whether production of *p*-cresol contributes to fitness *in vitro*, we performed co-culture assays with 630Δ*erm* and *hpdC*::CT cultured with a selection of intestinal commensal species, supplemented with exogenously added *p*-cresol. Brain heart infusion media with yeast extract (BHIS) was chosen for these co-culture experiments, as we have previously shown that intrinsic production of *p-*cresol under these conditions is negligible[22], therefore any observed effect could be attributed to the exogenously added *p-*cresol. We observed no difference in growth rate between 630Δ*erm* and *hpdC*::CT in these conditions [22]. To establish the comparable growth conditions, each species was normalised to the same starting optical density (OD_595_ 0.5) and starting CFU/ml was determined (S4 Table). The competitors were mixed in a 1:1 ratio at matched OD, and were grown for 24 hours in media supplemented with 0.05% (v/v) *p-*cresol. Viable counts for each species were determined by plating serial dilutions onto media supplemented with and without D-cycloserine and cefoxitin, facilitating differentiation between *C*. *difficile* and the competitor. When *C*. *difficile* 630Δ*erm* was grown in co-culture with *E*. *coli* in the absence of exogenous *p*-cresol, *E*. *coli* was the dominant organism, represented by a significantly higher CFU/ml than *C*. *difficile* (8:1 ratio of *E*. *coli* to *C*. *difficile*) (COV = 1.02, *p* = 0.003; Fig 2, S5 Table). However, when the medium was supplemented with exogenous *p*-cresol, the relative proportion of *C*. *difficile* increased to a ratio of 1:1, representing an 8-fold increase in the number of viable *C*. *difficile* (Fig 2A) (COV = -1.38, *p*<0.001). A similar profile was observed when *E*. *coli* was co-cultured with the *hpdC*::CT mutant (Fig 2B) (COV = -0.27, *p =* 0.882). This suggests that 630Δ*erm* and the *hpdC*::CT mutant displayed comparable fitness when grown in co-culture with *E*. *coli*. When *C*. *difficile* was grown in co-culture with a Gram-positive bacterium, *E*. *faecium*, *C*. *difficile* was significantly less abundant compared with the competitor (COV = 3.41, *p*<0.001). Here, we observed a ratio of 1:10 of *C*. *difficile* to *E*. *faecium* (Fig 2C & S5 Table). The growth dynamics of the *C*. *difficile hpdC*::CT mutant and *E*. *faecium* (Fig 2D) were also indistinguishable from the 630Δ*erm* grown in competition with *E*. *faecium* (COV = -0.33, *p* = 0.283). However, when the medium was supplemented with *p-*cresol, the relative proportion of *E*. *faecium* increased significantly (COV = 1.44, *p* = 0.010). This suggests that the growth conditions were more permissive for *E*. *faecium*. However, this was not the case for all Gram-positive species tested. The relative ratio in co-culture of *C*. *difficile* (630Δ*erm* and *hpdC*::CT mutant) to *L*. *fermentum* was not significantly altered by exogenous *p*-cresol (COV = -0.058, *p* = 0.818) (Fig 2E & 2F and S5 Table). These data indicate that *p*-cresol had a range of effects on growth dynamics depending on the Phylum of bacteria and their susceptibility to *p*-cresol.

### Intrinsic production of *p*-cresol gives *C*. *difficile* a competitive growth advantage *in vitro*

**Fig 3:**
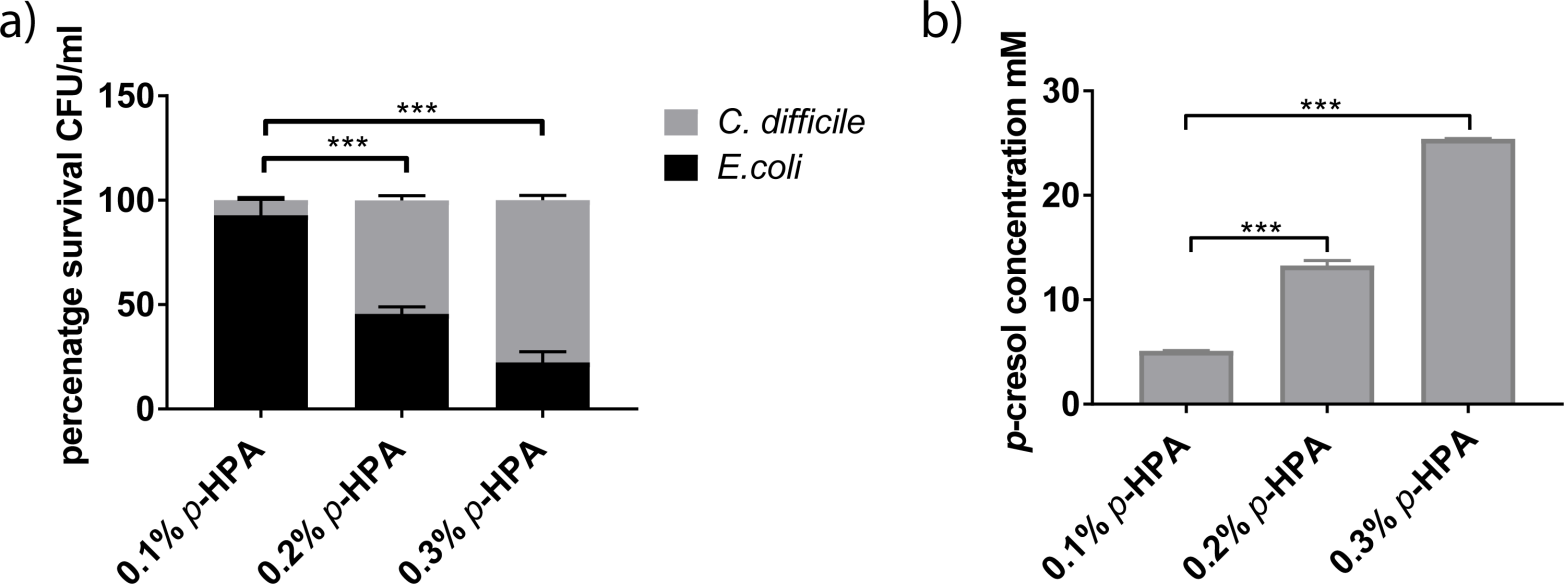
The effect of *p*-HPA supplementation on competitive co-culture of *C*. *difficile* with *E*. *coli* and detection of *p*-cresol production. a) A comparison of the percentage survival of *C*. *difficile* strain 630Δ*erm* and *E*. *coli* in competitive co-culture for 24 hours, performed in 0.1%, 0.2% and 0.3% *p-*HPA. Statistical difference in percentage survival was calculated using regression analysis taking strain and media into consideration ***p<0.001. b) HPLC quantification of *p*-cresol production in competitive co-culture of *C*. *difficile* strain 630Δ*erm* with *E*. *coli* in media supplemented with 0.1%, 0.2% and 0.3% *p-*HPA. Statistical difference in *p*-cresol production was calculated using a two tailed t-test with Welch’s correction, comparing individual *p*-cresol concentrations to the level produced in 0.1% *p*-HPA ***p<0.001.

We observed no difference in competitive fitness between wild type and *p-*cresol mutant when grown in BHIS supplemented with 0.5% (w/v) *p-*cresol. Therefore, we developed an additional *in vitro* competition assay to determine whether intrinsic *p-*cresol production by *C*. *difficile* conferred a competitive advantage over other intestinal commensal species. To achieve this we measured the growth rate of *E*. *coli* and *K*. *oxytoca* in monoculture and compared this to that of *C*. *difficile* (S1A Fig). Under these conditions, *C*. *difficile* reached exponential growth at a later time point than the other species tested. Furthermore, we have previously shown that *p-*cresol is detected in *C*. *difficile* cultures at around 4 hours (or OD_595_ 0.5) [22]. In order to limit the dominance of competitor species, and ensure optimal *p-*cresol production, we grew *C*. *difficile* to exponential phase (OD_595_ 0.6) before inoculating the medium with the competitor (at OD_595_ 0.05). We also supplemented the growth medium with *p-*HPA to drive production of *p-*cresol. To determine a concentration of *p*-HPA that resulted in inhibitory *p-*cresol production, competitive co-culture experiments with *C*. *difficile* and *E*.*coli* were performed in a range of *p-*HPA concentrations (0.1%, 0.2% or 0.3% (w/v)) (Fig 3). When the medium was supplemented with 0.1% (6.5 mM) *p*-HPA we observed a ratio of 13:1 (*E*. *coli*:*C*. *difficile*) (Fig 3A). When the concentration of *p*-HPA was increased to 0.2% (13.1 mM), we observed a significant difference (*p*<0.001) in the ratio of *E*. *coli*: *C*. *difficile* (1:1), compared to the ratio in 0.1% *p*-HPA (13:1)(Fig 3A). Further increasing the concentration of *p*-HPA to 0.3% (19.7 mM) resulted in culture conditions that favoured *C*. *difficile*, reflected by a ratio of 1:4 (*E*. *coli*:*C*. *difficile*) (*p*<0.001)(Fig 3A). Thus, we observed a positive correlation between the proportion of *p*-HPA supplemented in the growth medium and the survival of *C*. *difficile* compared to *E*. *coli* (Fig 3A). To determine whether this effect was linked to the level of *p-*cresol production, we quantified *p-*cresol in these culture supernatants by High Performance Liquid Chromatography (HPLC) (Fig 3B). Fig 3B demonstrates that increasing *p*-HPA concentration correlated with a significant increase in *p*-cresol production (*p*<0.001). We observed 25 ±0.04 mM *p-*cresol when the growth medium was supplemented with 0.3% *p*-HPA (Fig 3B).

**Fig 4:**
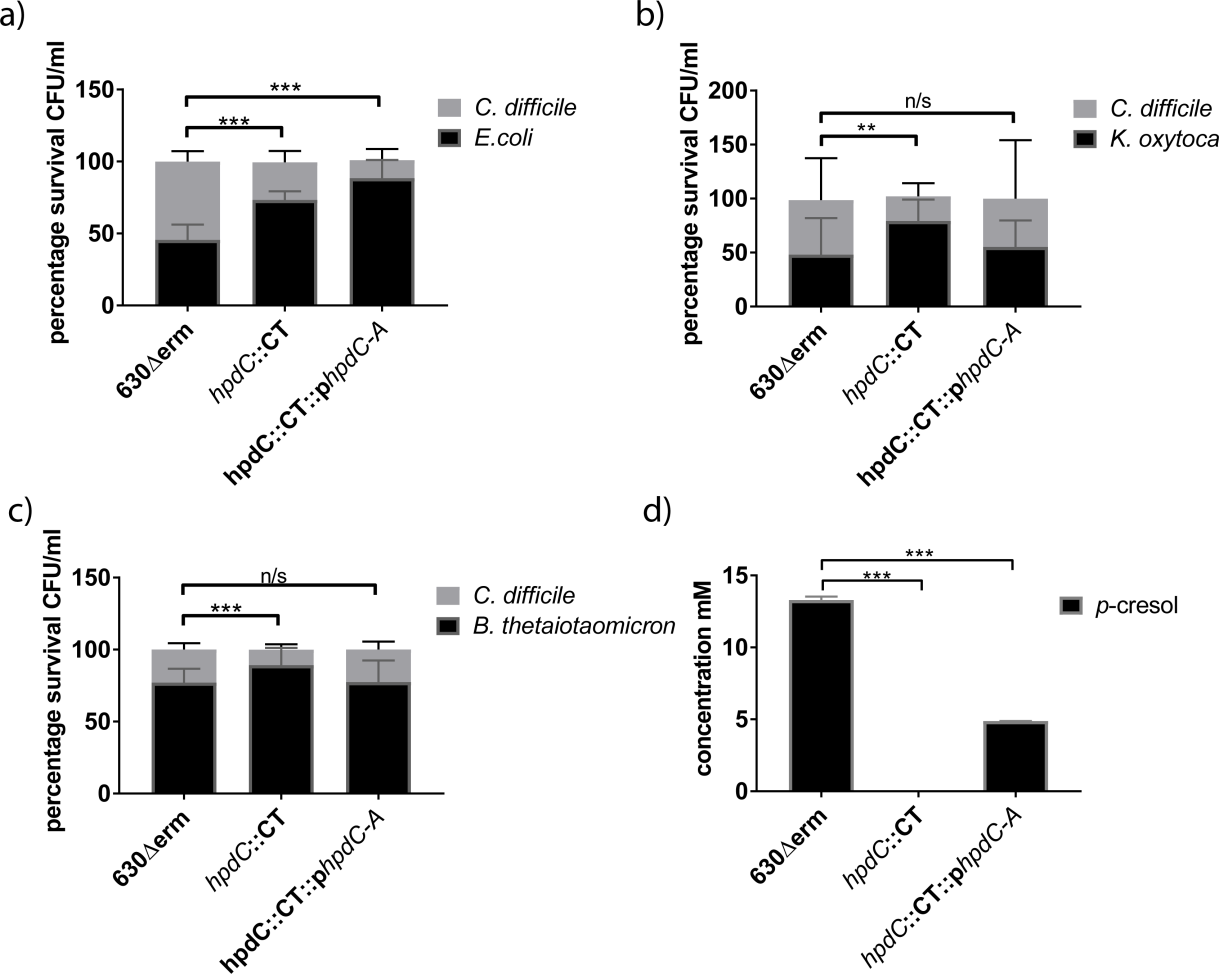
Competition co-culture assays in the presence of endogenous *p*-cresol. Relative fitness of *C*. *difficile* strains 630Δ*erm*, *hpdC*::CT and the complement *hpdC*::CT::p*hpdC-A* were grown in competitive co-culture for 24 hours with gut commensal species a) *E*.*coli*, b) *K*. *oxytoca* and c) *B*. *thetaiotaomicron*. The growth medium was supplemented with the intermediate in the *p*-cresol pathway, *p*HPA (at 0.2% v/v) and 50 ng/ml anhydrotetracycline to induce expression of *hpdCA*, encoded *in trans*. The relative representation of each strain was expressed as a percentage of the total CFU count. Error bars are representative of three independent replicates and show the variation across replicates and co-culture conditions. Regression analysis was used to determine significant differences in growth taking strain into consideration and marked ** *p*<0.01 and ***p<0.001. d) The concentration of *p*-cresol produced in the co-cultures of three independent replicates was quantified by HPLC and plotted in GraphPad Prism7. Statistical analysis was performed by Linear regression, significant differences in *p*-cresol production compared to 630Δ*erm* are indicated *** *p*<0.001.

Next, we investigated whether the *p-*cresol mutant displayed reduced fitness when grown in competitive co-culture with other gut competitor species. Furthermore, we constructed a complement by expressing the *hpdC* and *hpdA* genes from a tetracycline-inducible promoter using a plasmid based system (generating strain *hpdC*::CT::p*hpdCA*). We compared the growth of *C*. *difficile* strains 630Δ*erm*, *hpdC*::CT and the complement (*hpdC*::CT::p*hpdCA*), in competition with *E*. *coli*, *K*. *oxytoca* or *B*. *thetaiotaomicron* in media supplemented with 0.2% *p-*HPA (Fig 4). The number of viable counts for each species was determined as outlined above. When 630Δ*erm* was grown in co-culture with *E*. *coli*, we observed a 1:1 ratio of *C*. *difficile* to *E*. *coli* (Figs 4A & 3A). This was consistent with the co-culture assays supplemented with exogenous *p*-cresol (Fig 2). However, competitive co-culture between *hpdC*::CT and *E*. *coli* resulted in a decrease in the relative proportion of *C*. *difficile* to ca. 25% of the total culture (1:4, *C*. *difficile*:*E*. *coli*). This indicates that the mutant was significantly less viable than the wild type (COV = -1.06, *p*<0.001). 630Δ*erm* demonstrated comparable relative fitness in competitive co-culture with *K*. *oxytoca* (1:1 ratio) (Fig 3B). However, we observed proportionally fewer CFUs of 630Δ*erm* when grown in co-culture with *B*. *thetaiotaomicron* (1:4 ratio) (Fig 3C). By contrast, *hpdC*::CT displayed reduced fitness relative to 630Δ*erm* when grown in competition with both *K*. *oxytoca* (COV = -1.40, *p*<0.001) and *B*. *thetaiotaomicron* (COV = -0.79, *p* = 0.001). This fitness defect was restored when the complement was grown in competition with *K*. *oxytoca* and *B*. *thetaiotaomicron* (Fig 3). However, complementation of the *hpdC* mutation, did not restore *C*. *difficile* fitness to wild-type levels in competitive co-cultured with *E*. *coli*. Therefore, we quantified both *p*-cresol production and *p*-HPA utilisation by HPLC (Figs 4D, 3B & S2). Quantification of 630Δ*erm* supernatants grown in both monoculture and competitive co-culture supplemented with 0.2% *p*-HPA revealed an average *p-*cresol concentration of 13.3 ±0.1 mM. In contrast, the concentration in supernatants of the complemented mutant (at 0.2% *p*-HPA) was only 4.8 ±0.2 mM, representing a significant 2.7 fold reduction (*p*<0.01). Therefore, we conclude that under competitive co-culture conditions, 4.8 ±0.2 mM *p*-cresol was sufficient to have a deleterious effect on the growth of *K*. *oxytoca* and *B*. *thetaiotaomicron*, but not on *E*. *coli*. Increasing the concentration of the transcriptional inducer (anhydrotetracycline) and *p*-HPA resulted in increased *p*-cresol production (from 4.8 ±0.2 mM to 15.6 ±3.9 mM) by the complement and restoration of the phenotype (S2 Fig). Here, the level of *p*-cresol production directly correlated with the concentration of the transcriptional inducer (S2C and S2D Fig). As expected, this suggests that complementation was more greatly influenced by transcript expression rather than availability of the *p-*HPA precursor. Furthermore, we observed no difference in growth rate between the three *C*. *difficile* strains at any tested concentration of anhydrotetracycline (S1 Fig). Taken together, these data suggest that production of *p-*cresol by *C*. *difficile* confers a competitive growth advantage over susceptible bacterial species (specifically, Gram-negative species) under our *in vitro* conditions.

### Metabolic profiling of *C*. *difficile* and the intestinal commensal species

**Fig 5:**
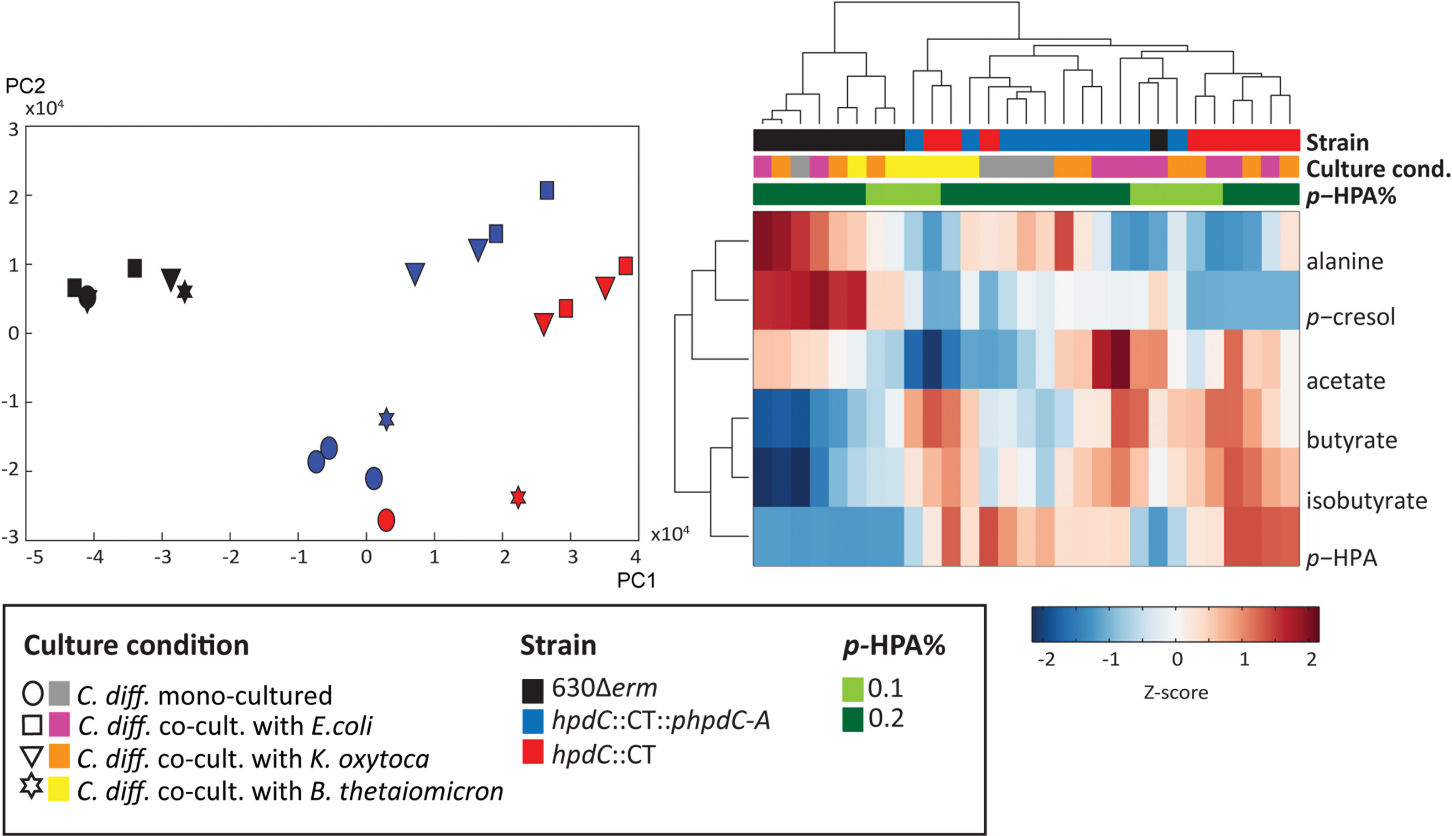
Metabolic profiling of *C*. *difficile* in monoculture and competitive co-culture with intestinal commensal species. a) Principle component analysis (PCA) score plot based on the ^1^H NMR spectra of the three *C*. *difficile* strains (630Δ*erm* (black), *hpdC*::CT (red) and the complemented mutant (*hpdC*::CT::p*hpdC-A*) in (blue), grown in monoculture (circle symbol) or in competitive co-culture with intestinal commensal species, *E*. *coli* (square), *K*. *oxytoca* (triangle) and *B*. *thetaiotaomicron* (star), in BHIS media supplemented with 0.2% *p-*HPA. b) Metabolite clustergram represented as a heatmap of *Z*-scores of the ^1^H NMR peak integrals, derived from the quantity of six main metabolites driving the separation between the samples (observed on the loading plots of the model) and a dendrogram showing the hierarchical clustering of the samples. The culture conditions, bacterial strains and *p*-HPA concentration are colour coded in a key on the heatmap.

To further understand the effect of *p-*cresol production on the interaction between *C*. *difficile* and these intestinal species, we characterised the metabolic content of both mono-culture and competitive co-culture supernatants using ^1^H nuclear magnetic resonance (NMR) spectroscopy. We analysed the culture supernatants described in Fig 4 and performed principal component analysis (PCA) to identify metabolic variation across the profiles. The scores plot from the PCA model comparing all profiles showed that the largest variation in the metabolic data (Principal Component 1 (PC1)) was between the *C*. *difficile* strain 630Δ*erm* samples and those from the *hpdC*::*CT* strain (Fig 5A). The metabolic profiles from the complement samples clustered between 630Δ*erm* and *hpdC* mutant samples. The loadings for PC1 describe the metabolites varying between the strains. This indicated that culture supernatants from 630Δ*erm* contained significantly greater amounts of *p*-cresol and alanine compared to the other strains, but lower amounts of *p*-HPA, butyrate and isobutyrate (Fig 5B). This was consistent with the notion that *p*-HPA is being depleted in order to produce *p-*cresol. The 630Δ*erm* samples clustered together regardless of whether the bacteria were grown in mono-culture or co-cultured with *E*. *coli*, *K*. *oxytoca* or *B*. *thetaiotaomicron*. In contrast, *hpdC*::CT and complement (*hpdC*::CT::p*hpdC-A*) strains were separated in the second principal component (PC2) based on the competitive co-culture conditions. The loadings for PC2 indicated that the mono-cultured *C*. *difficile* and *B*. *thetaiotaomicron* competitive co-culture samples contained lower amounts of acetate compared to the competitive co-cultures from *E*.*coli* and *K*. *oxytoca*. This metabolic variation between strains and competitive co-culture conditions is summarised in the clustergram shown in Fig 5B, which was constructed from the *Z*-scores of ^1^H NMR peak integrals measured for each metabolite across all samples. The dendrogram shows that the 630Δ*erm* metabolic profiles clearly cluster away from those of the other two strains (Fig 5) and the variation in *p*-cresol production between samples is apparent in the ^1^H NMR spectrum (S3 Fig). The dendrogram also showed that *p*-cresol and alanine clustered together as did butyrate and isobutyrate (Fig 5B). We also assessed the effect of altered *p*-HPA concentration on metabolic profiles. The PCA of metabolites produced in media supplemented with 0.1% and 0.2% *p-*HPA demonstrated no clear metabolic variation between these samples. However, we did observe clustering within the 0.1% *p-*HPA samples, driven by increased *p*-cresol and alanine (S4 Fig). These data suggest that under these growth conditions *p-*cresol is one of the most abundant metabolites in culture supernatants. This is reflected by both metabolic profiling and HPLC quantification, which correlates to *p*-cresol susceptibility observed in both competitive co-culture and monoculture of Gram-negative bacteria.

### Mouse relapse model of *C*. *difficile* infection

**Fig 6:**
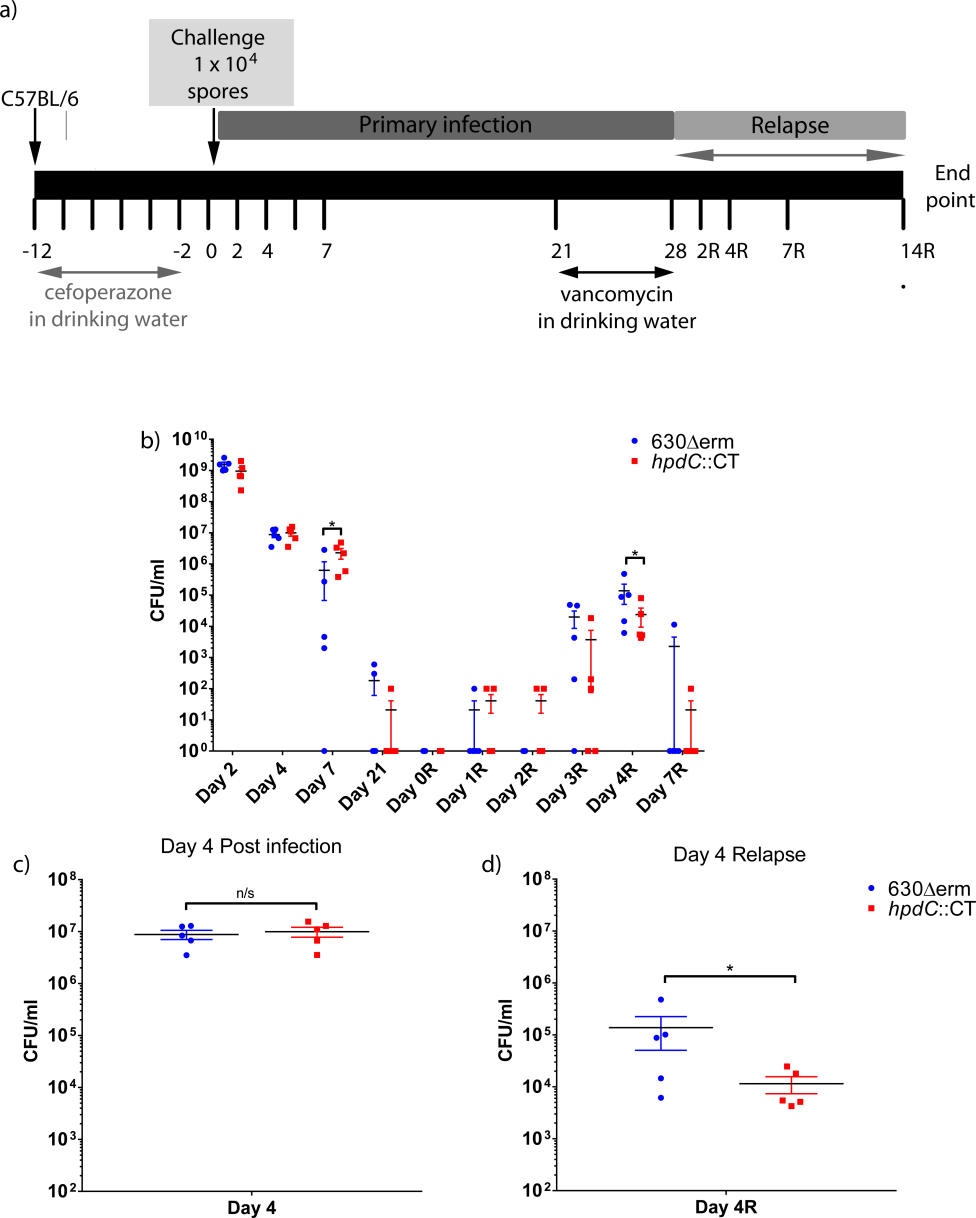
Infection of mice with 630Δ*erm* and *hpdC*::CT. a) A schematic of the *C*. *difficile* relapse infection in C57BL/6 mice. b) CFU counts enumerated from the faeces of individually caged animals (n = 5) infected with either wild-type *C*. *difficile* strain (630Δ*erm*) or the corresponding *p*-cresol null mutant (*hpdC*::CT) over the duration of the primary infection (days 1–21) and relapse (days 1R-7R). c) CFU enumerated from the faeces day 4 post-infection and d) day 4 relapse. Mann Whitney U test was used to calculate significant differences between infection with wild-type *C*. *difficile* and the *hpdC* mutant marked *p<0.05. Graphs were produced in GraphPad Prism 6.0.

Our results have demonstrated that *p-*cresol production confers a fitness advantage over discrete bacterial species *in vitro*. Therefore, we sought to determine whether this was also true *in vivo*. Individually caged C57BL/6 mice were infected in parallel with 1x10^4^ spores of *C*. *difficile* strain 630Δ*erm* (*n* = 5) or the *hpdC*::CT mutant (*n* = 5) and compared to uninfected naïve control mice (*n* = 5), in a relapse model of infection. Mice were given cefoperazone in their drinking water for 10 days to stimulate gut dysbiosis, before infection by oral gavage with *C*. *difficile* spores (Fig 6A)[23, 24]. Stool samples were collected throughout the experiment for analysis. Twenty-eight days post-infection, mice were treated with vancomycin in their drinking water for 7 days to encourage recurrence of infection (Fig 6A). Infection was monitored by enumeration of spores isolated from faeces on *C*. *difficile* selective media (Fig 6B). We observed no significant difference in the number of spores recovered from faeces of mice infected with either 630Δ*erm* or *hpdC*::CT following cefoperazone treatment, indicating that the *hpdC*::CT mutant and 630Δ*erm* were equally competent at initial colonisation (Fig 6C). This was consistent with the notion that these strains demonstrate similar resistance to cefoperazone and vancomycin (S6 Table) and no differences in sporulation *in vitro* (S5 Fig). However, at day 7 post-infection we observed a modest difference in colonisation, represented by significantly fewer 630Δ*erm* CFUs compared to the *hpdC*::CT mutant (*p*<0.05). Relapse was detected by enumeration of *C*. *difficile* spores post-vancomycin treatment. Three days following removal of vancomycin (D3R), spores were detected in all but one 630Δ*erm* infected mouse and three out of five *hpdC*::CT mutant infected mice. By day 4 post-relapse (D4R) *C*. *difficile* spores were recovered from the faeces of all mice and we observed a significant reduction (*p*<0.05) in the number of spores recovered from the faeces of mice infected with the *hpdC*::CT mutant relative to 630Δ*erm* infected mice (Fig 6D). Both infections followed a broadly comparable progression, however, we observed modest but significant differences in the number of spores recovered both pre- and post- relapse between 630Δ*erm* infected and *hpdC*::CT infected mice at discrete time points. Interestingly during relapse, the spore density remained lower in *hpdC*::CT compared to 630Δ*erm*, despite an initially higher CFU at D7 post-infection, indicating that these differences are a result of *in vivo* fitness.

### Composition of the intestinal microbiota

**Fig 7:**
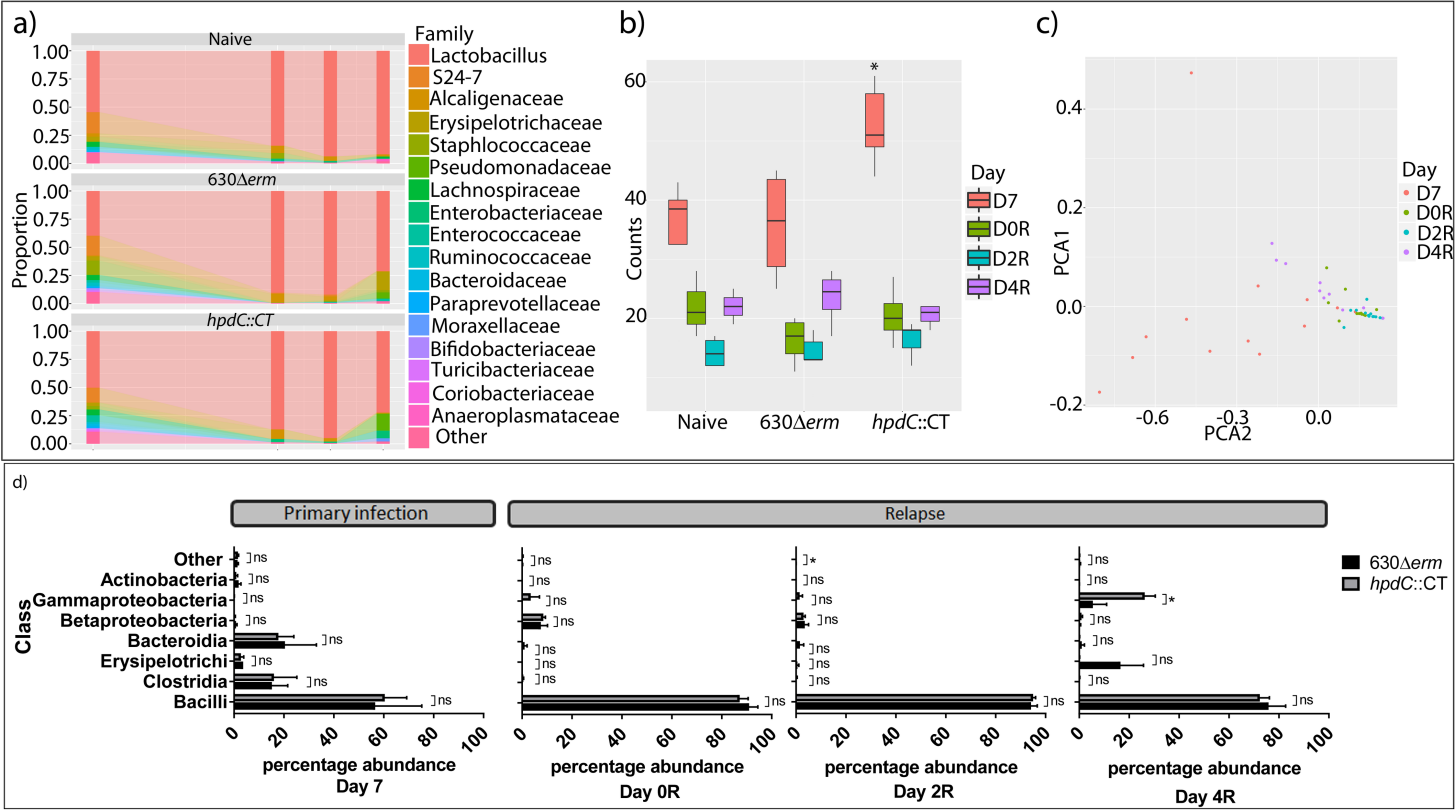
16S relative abundance. The diversity of the intestinal microbiota was assessed using 16S rRNA sequencing to determine the bacterial operational taxonomic units present during a relapse model of *C*. *difficile* infection. a) Composition of the intestinal microbiota, at the Family level, between wild type *C*. *difficile* 630Δ*erm* and *p-*cresol mutant (*hpdC*::CT) infected mice and the naïve control at day 7 post-infection, day 0, day 2 and day 4 post relapse. b) Box and whisker plots displaying the total number of operational taxonomic units (OTUs) at the Family level. Statistically significant differences between the diversity of OTU’s are labelled * *p*<0.05 (ANOVA). c) Principle component analysis of the microbial diversity at the Family level. Each dot represents a single mouse at a given time point, colour-coded for day 7 post-infection, and days 0, day 2 and day 4 post-relapse. d) The relative prevalence of bacterial Class in wild type *C*. *difficile* and *hpdC*::CT infected mice at day 7 post-infection, day 0, day 2 and day 4 post relapse. Statistical analysis was performed in Stata15 by regression analysis taking strain and bacterial class into consideration * *p*<0.05.

Given that the *hpdC*::CT mutant displayed an altered colonisation profile during relapse and that *p-*cresol displays bacteriostatic properties against a number of species, this led us to postulate that production of *p-*cresol may alter the composition of the intestinal microbiota in such a way that favoured *C*. *difficile* re-colonisation. We isolated bacterial DNA from four key time points during the relapse model of CDI; day 7 post-cefoperazone treatment, immediately upon cessation of vancomycin treatment (D0R), day 2 post-relapse (D2R) and day 4 post-relapse (D4R), when all the mice were colonised with *C*. *difficile* (1.6 x 10^6^ WT and 2.3 x 10^5^ *hpdC*::CT mutant spores/g faeces). To assess the community structure of the microbiota, 16S rRNA sequencing was performed by sequencing the V5-V7 region of 16S rRNA gene. The data was grouped with distance-based similarity of 97% into operational taxonomic units (OTUs), using Greengenes and associated summaries and diversity analyses were performed in QIIME. Consistent with previous studies, the microbiota of cefoperazone-treated mice was dominated by Lactobacillaceae (Fig 7A and S7 Table)[24–26], which comprised 39.7% (mean relative abundance) of the total microbiota in 630Δ*erm* infected mice. The microbiota was also populated by Bacteroidetes, including members of the S24-7 (an uncultured commensal of homeothermic animals[27]) (17.6%) and Paraprevotellaceae (1.2%) families. Furthermore, Firmicutes, including Staphylococcaceae (12.75%), other Clostridiales (7.2%), Lachnospiraceae (4.2%), Erysipelotrichaceae (3.7%), Ruminococcaceae (2.9%), Enterococcaceae (2.2%), Turicibacteraceae (1.2%), and Actinobacteria including Bifidobacteriaceae (1.9%) (Fig 7A) also contributed to the microbiota composition. However, animals infected with the *hpdC*::CT mutant demonstrated a significant increase in microbial diversity at D7 (ANOVA *p*<0.05), compared to 630Δ*erm* infected and naïve mice (Fig 7B and S7 Table), which is also upheld with an ANOSIM population analysis *p*<0.05 (S6 Fig). Consistent with the notion that *p*-cresol prevents outgrowth of Proteobacteria, the majority of the families that were only present in the *hpdC* mutant infected animals were members of the Proteobacteria Phylum (S7 Table), albeit at low abundance.

Treatment with vancomycin significantly reduced diversity of both uninfected (naïve) and infected mice (630Δ*erm* and *hpdC*::CT) (Fig 7B & 7C), resulting in a dramatic increase in the relative representation of Lactobacillaceae, specifically the *Lactobacillus* genus, which constituted ≥85% of the microbiota of all the mice examined (90% 630Δ*erm*, 87% *hpdC*::CT mutant and 85% naïve at D0R). Principle component analysis demonstrated clustering of D0 and D2 post relapse (Fig 7C). At D2R, the diversity of the microbiota remained low. At D4R, partial recovery of the microbial diversity was observed (Fig 7A and 7B), which coincided with the detection of *C*. *difficile* spore in faeces (Fig 6D). At D4R there were distinct differences in population composition in the intestinal bacteria of these animals (Fig 7D). We observed an increase in spread on the PCA plot at D4R (Fig 7C), compared to the clustering observed at D0R and D2R. Although the mean relative proportions of Lactobacillaceae were similar (73.9% in 630Δ*erm* and 72.3% in the *hpdC*::CT mutant infected animals), there were clear differences in the representation of other taxa, including other Firmicutes, Proteobacteria and Bacteroidetes (Fig 7D). In 630Δ*erm* infected mice there was an increase in representation of Firmicutes from the Erysipelotrichales (16.5% 630Δ*erm* and 0.1% *hpdC*::CT mutant), Bacillales (1.9% 630Δ*erm* and 0% *hpdC*::CT mutant) and Clostridiales orders, and the Bacteroidetes (Fig 7D). Conversely, in the *p*-cresol mutant infected mice, we observed an increase in the representation of Proteobacteria, specifically, the Gammaproteobacteria of the Pseudomonadales (5.5% 630Δ*erm* and 18% *hpdC*::CT mutant) and Enterobacteriales (0% 630Δ*erm*, 6.75% *hpdC*::CT mutant) order, and the Betaproteobacteria of the Burkholderiales order (Fig 7D). Consistent with the notion that Gram-negative species were more susceptible to the effects of *p-*cresol, Gammaproteobacteria formed 26.2% of the total microbiome in *hpdC*::CT infected animals D4R, compared with 5.5% in 630Δ*erm* infected mice (COV = 9.37, *p* = 0.023), suggesting that *p*-cresol may inhibit their outgrowth following treatment with vancomycin.

### Metabolic profiling of the mouse model of CDI

**Fig 8:**
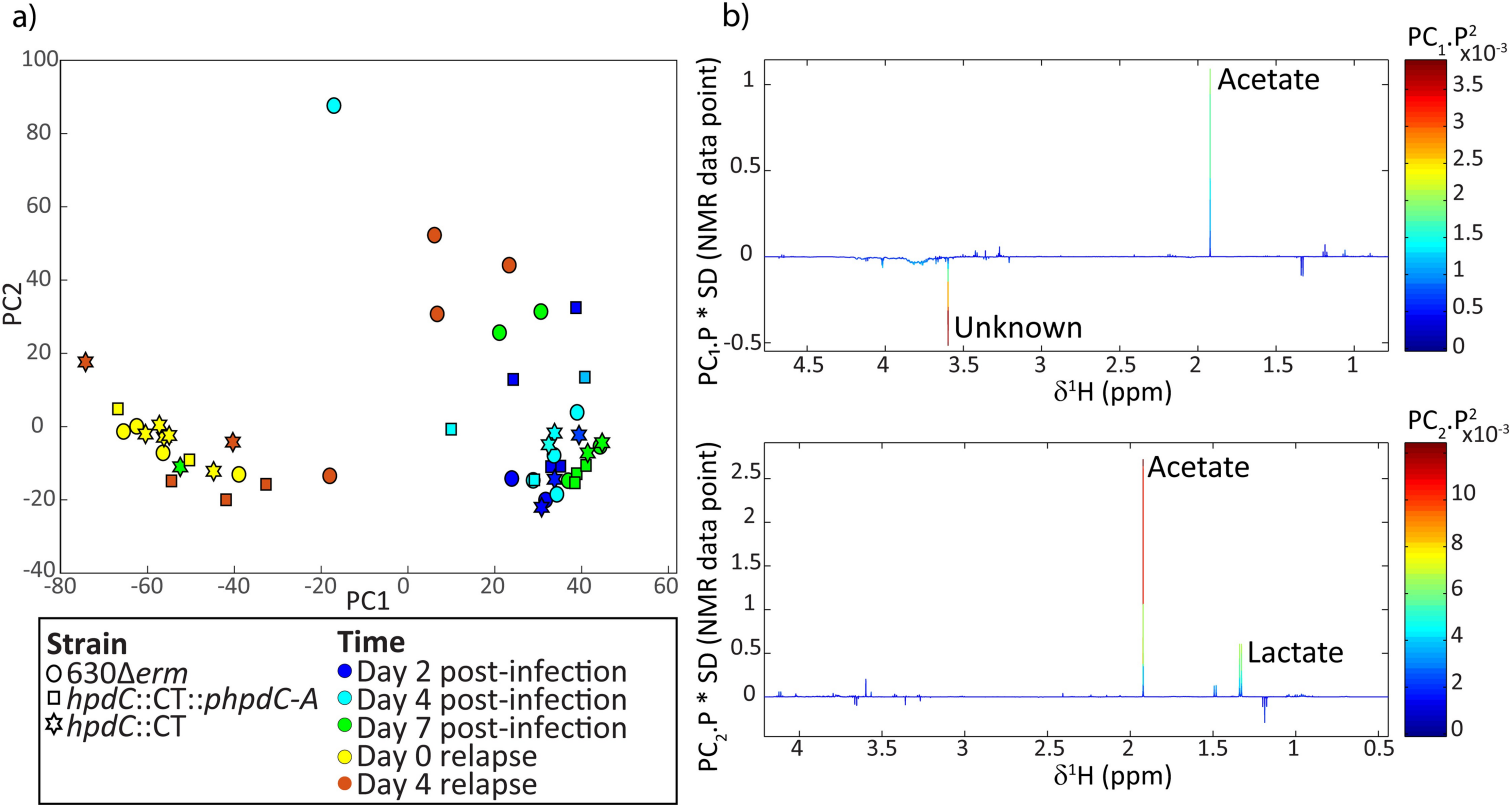
Metabolic profiling of the *in vivo* mouse relapse model of CDI. ^1^H NMR spectroscopy was used to investigate metabolic differences between the individually caged mice infected with *C*. *difficile* strain 630Δ*erm*, *hpdC*::CT or uninfected naïve mice. a) Principle component analysis of the metabolite diversity. Each dot represents a single sample at a given time point. A symbol code was used for the strains 630Δ*erm* (circle), *hpdC*::CT (square) and naïve mice (star), and a colour code was used to represent the different time points, Day 2 post-infection (dark blue), Day 4 post-infection (light blue), Day 7 post-infection (green), Day 0 relapse (yellow) and Day 4 relapse (orange). b) Loading plots of the PCA model, representing the metabolites driving the distribution of the samples along principal component 1 (PC 1) and principal component 2 (PC 2).

Our data suggest that *p-*cresol production by *C*. *difficile* influenced the composition of the mouse faecal microbiota. Therefore, we investigated whether these differences resulted in an altered biochemical profile. Stool samples collected throughout the duration of the initial mouse infection (at day 2 (D2), 4 (D4) and 7 (D7)) and during relapse (at days 0 (D0R) and 4 (D4R)) were analysed using ^1^H NMR spectroscopy (Fig 8). The PCA scores plot identified biochemical variation between the faecal profiles collected D2-D7 versus D0R-D4R in the control mice and those infected with the *hpdC*::CT mutant. The D2-D7 samples contained greater acetate compared to the relapse time points, and lower amounts of an unknown metabolite (δ 3.59, singlet). Vancomycin induced perturbations in the metabolic activity of the intestinal bacteria are likely to underlie these changes[28].

The faecal profiles from mice infected with the 630Δ*erm* strain showed similar metabolic alterations to the control mice and those infected with the mutant strain during the initial infection (D2-D7). However, the response was different 4 days after relapse. At D4R, the faecal profiles from mice infected with the 630Δ*erm* strain were more variable than the uninfected mice and those infected with the mutant strain and were similar in composition to the initial infection profiles (Fig 8).

### Effect of *p*-cresol on viability and diversity of the human microbiome

**Fig 9:**
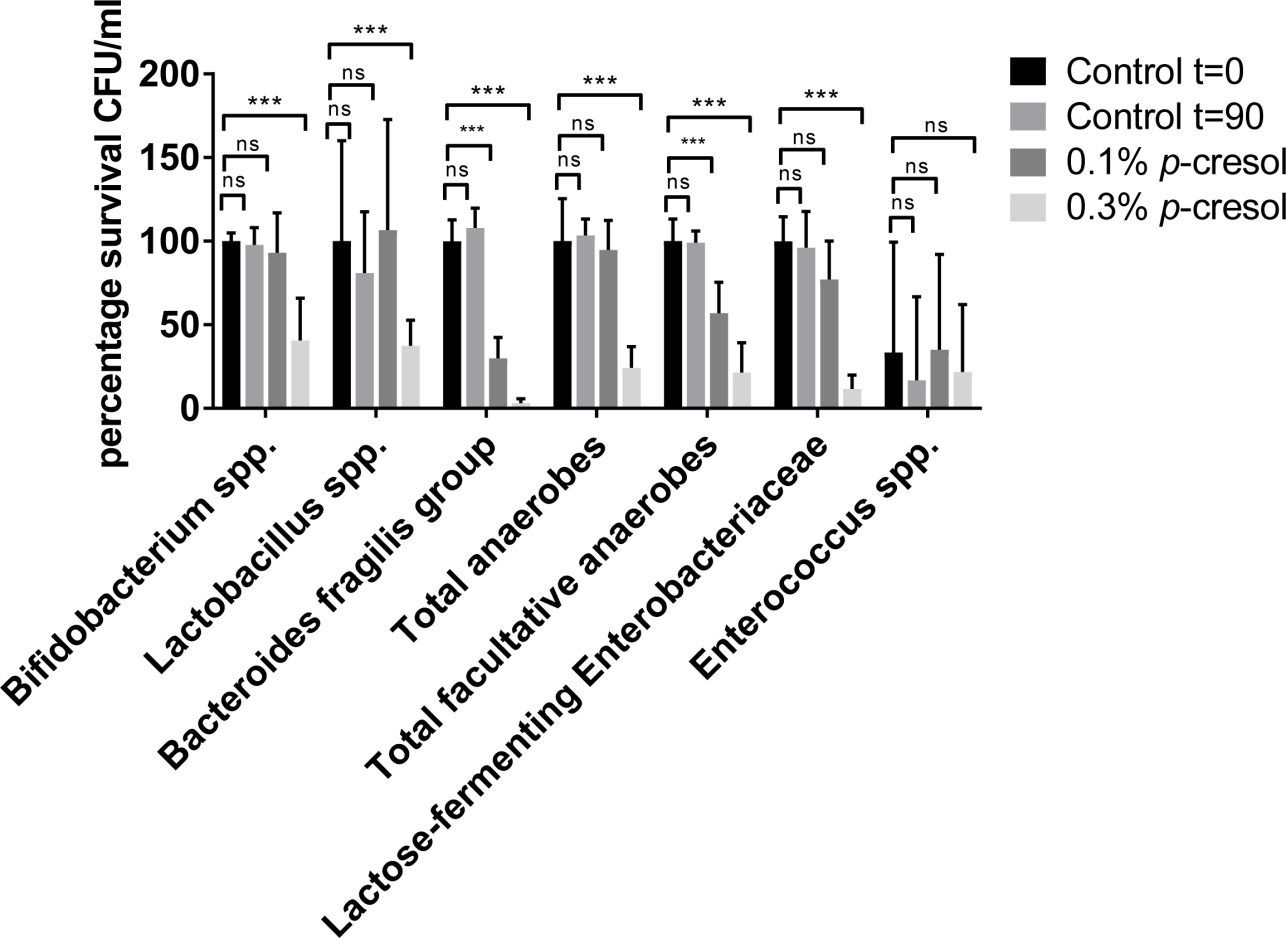
Percentage survival of bacterial phyla from healthy human faecal slurry treated with *p*-cresol. Healthy human donor stool samples were emulsified and treated with exogenously added *p*-cresol (0.1% or 0.3% v/v) for 90 minutes and the CFU counts were expressed as percentage survival relative to the CFU of the PBS control at t = 0. Faecal emulsions were plated onto differentially selective agar to determine the survival of endogenous intestinal bacteria to *p*-cresol stress. Error bars are representative of triplicate experiments. Statistical analysis was performed by linear regression to determine the correlation between survival in 0.1% and 0.3% (v/v) *p*-cresol relative to the PBS control. Significant differences are indicated with ***p<0.001. Error bars are Standard error of the Mean (SEM).

We have shown that *p-*cresol production has deleterious effects on the outgrowth of Gram-negative bacterial species both *in vitro* (Figs 1–4), and in an *in vivo* mouse infection model (Fig 7). Thus, we sought to determine the effect of exogenously added *p*-cresol on biodiversity of the human microbiome. Healthy human stool samples were taken from donors ranging from 60–65 years old, who had not received antibiotic treatment in the last 3 months, eliminating possible perturbations by antibiotic therapy. We measured the effect that exogenously-added *p*-cresol (at 0.1% and 0.3%) had on faecal microbiota, compared to a Phosphate Buffered Saline (PBS) control. Differential plating revealed that the facultative anaerobes were particularly sensitive to *p*-cresol at both 0.1% (COV = -0.61, *p* = 0.006) and 0.3% (COV = -1.82, *p*<0.001), represented by a significant reduction in viable counts (Fig 9). The *Bacteroides fragilis* group was also significantly reduced after exposure to both 0.1% (COV = -1.29, *p* = 0.009) and 0.3% (COV = -4.39, *p*<0.001) *p*-cresol (Fig 9). The total anaerobes and lactose-fermenting *Enterobacteriaceae* were also significantly reduced after exposure to 0.3% *p*-cresol (COV = -1.48, *p*<0.001, COV = -2.36, *p*<0.001, respectively) (Fig 9). Consistent with the mouse model of CDI, *p*-cresol at 0.1% had a limited effect on the survival of *Lactobacillus* (COV = -0.045, *p* = 0.890) and *Bifidobacterium* species (COV = -0.100, *p* = 0.642). However, a significant decrease in survival was observed for both groups when they were incubated in 0.3% *p-*cresol (*p*<0.01). In line with our *in vitro* co-culture data (Fig 2), *Enterococcus* species present in human faecal samples were not adversely affected by the addition of *p*-cresol (Fig 8), even at the highest concentrations tested (COV = 0.48, *p* = 0.873).

### *p*-cresol compromises the integrity of Gram-negative cell envelope

**Fig 10:**
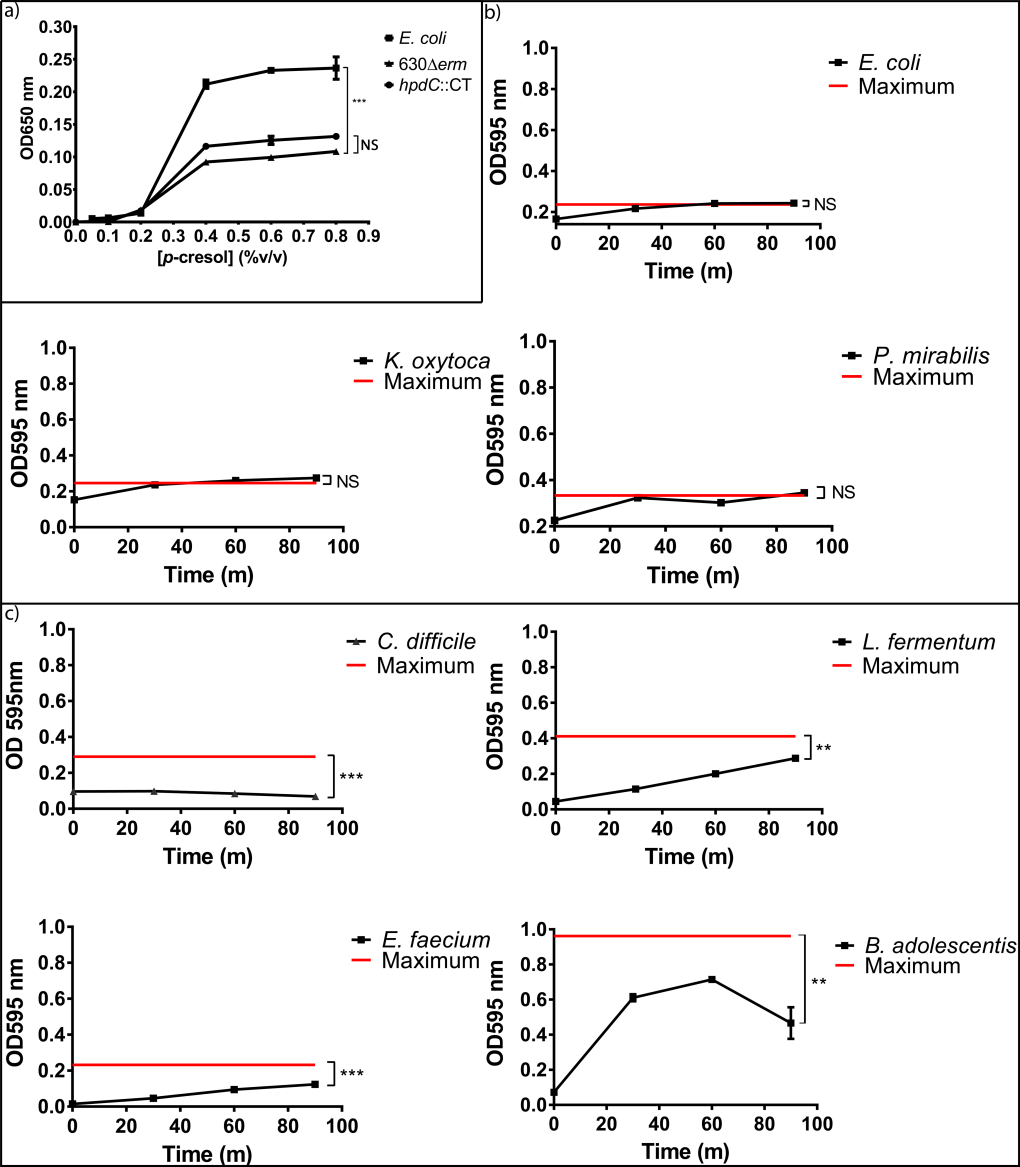
Phosphate release in Gram-positive and Gram-negative common gut commensal species. The release of intracellular phosphate was determined spectroscopically (OD_650_) using malachite green and ammonium molybdate from: a) *C*. *difficile* 630Δerm, *hpdC*::CT and *E*. *coli* stationary phase cultures in various concentrations of *p*-cresol. Significant differences in phosphate release compared to 630Δ*erm* were calculated taking concentration into account using linear regression and are marked *** *p*<0.001. b) phosphate relapse was measured from Gram-negative common gut commensals and c) phosphate relapse was measured from Gram-positive common gut commensals following incubation with 0.3% (v/v) *p-*cresol over a 90 minute time period. Red line indicates maximum phosphate release determined by boiling a comparable cell suspension. Curves and error bars are representative of three independent replicates. Significant differences in phosphate release from the maximum were calculated using regression analysis taking time points into consideration, significant differences are marked **p<0.01, ***p<0.001. Error bars are SEM.

Given that we observed a clear distinction in the nature of species that displayed tolerance to *p-*cresol, we reasoned that the cell envelope would be an obvious target for its mode of action. Phenolic compounds that target membranes typically induce a rapid loss of low molecular weight compounds from within the cell as a result of increased membrane permeability[29–31]. Thus, we used the release of inorganic phosphate (Pi) as a metric for determining membrane integrity in the presence of *p-*cresol. Initially, we compared the release of phosphate from *E*. *coli* and *C*. *difficile* strains (630Δ*erm* and *hpdC*::CT) in increasing concentrations of *p-*cresol (Fig 10A). We observed a significant increase in the amount of phosphate released by *E*. *coli* compared to *C*. *difficile* (COV = 0.868, *p* = 0.005). Only 16% of the total intracellular phosphate of *C*. *difficile* was released upon contact with *p-*cresol. Furthermore, the *hpdC*::CT mutant displayed a similar phosphate release profile to 630Δ*erm C*. *difficile* (COV = 0.201, *p* = 0.444), which was not significantly different (Fig 10A). This indicates that disruption of *p*-cresol production had little bearing on *p*-cresol tolerance, under these conditions. Our data suggests that cells can tolerate *p*-cresol up to a threshold level (~0.4% v/v), after which the amount of phosphate released becomes saturated. Therefore, we selected a concentration of 0.3% (v/v) to determine phosphate release over time in a selection of Gram-positive and Gram-negative gut bacteria. Membrane integrity was measured in the presence of *p-*cresol by comparing the amount of *p*-cresol induced phosphate release, to the total intracellular phosphate, which was determined by boiling cell suspensions for 15 minutes. Fig 10 demonstrates that species with a Gram-positive cell envelope display greater tolerance to *p-*cresol than Gram-negative species, represented by significantly less phosphate release (COV = -2.478, *p*<0.001), (Lactobacillales: *E*. *faecium* (*p* = 0.005) and *L*. *fermentum* (*p* = 0.003), the Bifidobacteriales: *B*. *adolescentis* (*p =* 0.01) and the Clostridiales: *C*. *difficile* (*p*<0.01)) corroborating previous observations. Conversely, the Gram-negative Gammaproteobacteria: *P*. *mirabilis*, *E*. *coli* and *K*. *oxytoca* released their total intracellular pool of phosphate over the course of the assay. *P*. *mirabilis and K*. *oxytoca* released 68% and 60% of their total phosphate respectively immediately upon contact with *p-*cresol (Fig 10B). Both species released >90% of their total phosphate pool following 30 minutes contact with *p-*cresol. In contrast, no Gram-positive species analysed released their total intracellular phosphate pool over the course of the assay (Fig 10C). However, *B*. *adolescentis* released 63% of its total phosphate at 30 minutes compared to 20% for *E*. *faecium*, 27% for *L*. *fermentum* and 33% for *C*. *difficile*, indicating that *B*. *adolescentis* is more sensitive to *p*-cresol than other Gram-positive species. Prolonged exposure to *p-*cresol resulted in other Gram-positive species releasing a greater portion of their intracellular pool of phosphate, however, the level of Pi released by *C*. *difficile* never exceeded its initial level of release (Fig 10). In conclusion, our data demonstrate a clear correlation between bacterial cell envelope structure and susceptibility to *p-*cresol.

## Discussion

The indigenous microbiota has been shown to form an ecological barrier that prevents the ingress of pathogenic bacteria such as *C*. *difficile* [32]. However, the specific components of the intestinal microbiota that facilitate colonisation resistance are only recently becoming clear [5–7, 25, 33–36]. Both the treatment with broad-spectrum antibiotics and the availability of specific metabolites has been shown to play a role in the expansion of particular bacterial species within the human microbiota [37, 38]. Here, we present compelling evidence that *C*. *difficile* may directly modify the intestinal microbiota through production of *p*-cresol. We demonstrate that *C*. *difficile* displays a greater degree of tolerance to *p-*cresol compared to other common intestinal species, including the Gammaproteobacteria: *E*. *coli*, *K*. *oxytoca* and *P*. *mirabilis*, as well as the Bacteroidetes, *B*. *thethaiotaomicron*. We show that these bacterial species are susceptible to the effects of both endogenous and exogenous *p-*cresol, which was reflected in reductions of viable counts when these intestinal microbiota species were grown in competitive co-culture with *C*. *difficile*. Using a plasmid based complementation system to restore the expression of the *p*-HPA decarboxylase, we have shown that *p*-cresol production by *C*. *difficile* must exceed 5 mM to elicit a significant alteration in competitive growth dynamics. We have shown that *C*. *difficile* is able to utilise all the available *p-*HPA supplemented in the growth medium, which results in the production of up to 25 ±0.04 mM *p*-cresol *in vitro* (Fig 3), which is 1000-fold more than the amount of *p*-cresol produced from tyrosine metabolism by other organisms cultured from the intestinal microbiota (range 0.06–1.95 μg/ml)[11]. There is evidence that *p-*HPA is present in the human colon and detected in healthy human stool samples at 19 μM[15], therefore *C*. *difficile* can potentially utilise free tyrosine and *p-*HPA to produce *p*-cresol *in vivo*.

We expanded our investigation of the influence of *p-*cresol on the growth of other bacterial species, to identify other metabolites influencing growth. In particular, alanine, *p*-cresol, acetate, butyrate, isobutyrate and *p*-HPA were the six main metabolites that were differentially modulated in mono-culture and co-culture of *C*. *difficile* with intestinal bacteria. The abundance of these metabolites *in vitro* was altered in the presence of the *p*-cresol mutant compared to *C*. *difficile* strain 630Δ*erm*. Acetate and butyrate are the most common end products of fermentation in the gut[39]. *C*. *difficile* can use amino acids as the sole energy source via Stickland fermentation, in which amino acid acceptors (such as glycine, proline and hydroxyproline) are reduced in a paired metabolism with electron donors (such as leucine, isoleucine or alanine). This can result in the conversion of alanine to acetate [40]. However, in *C*. *difficile* monoculture, we did not observe an inverse association between acetate and alanine (Fig 5B), suggesting that *C*. *difficile* is not utilising alanine in stickland fermentation under nutrient rich conditions (in BHIS media). This suggests that the competitor species, may have been responsible for the increased utilisation of alanine in co-culture with the *p*-cresol mutant and complement, where the competitor is more abundant. The reduction of the Stickland acceptors glycine and proline in *C*. *sticklandii and C*. *difficile* requires two selenium dependant reductases, glycine reductase and D-proline reductase [40, 41], highlighting the importance of selenium in growth and metabolism in *C*. *difficile*, particularly in glycine reduction and selenocysteine production [40, 42]. Nutrient availability has been linked to virulence in *C*. *difficile* in a number of different ways, via the global transcriptional regulators CodY, CcpA, PrdR and Rex, which are involved in overlapping cellular processes including toxin production, amino acid biosynthesis, stickland fermentation, nutrient transport, fermentation and cell membrane components[43]. The hypervirulent *C*. *difficile* strains (RT027 and RT078) have also developed the ability to metabolise low concentrations of trehalose, via acquisition of a single point mutation in the trehalose repressor (*treA*), which increases virulence of these ribotypes *in vivo*[44]. *C*. *difficile* is also capable of utilising ethanolamine as a carbon source[45] and the ethanolamine genes are upregulated *in vivo* in the presence of *B*. *thetaiotaomicron* when animals were fed on a standard polysaccharide diet [38].

Cysteine is involved in amino acid and energy metabolism in *C*. *difficile* [46], modulating processes such as carbon transfer, electron transport, butyric acid and butanol production. Cysteine results in increased levels of intracellular tyrosine [47]. Cysteine also down-regulated *4p*-hydroxyphenylacetate-3-hydroxylase [48], which may reduce *p*-HPA availability for the *p*-HPA decarboxylase and thus would decrease flux to *p*-cresol. Therefore, cysteine-regulated pathways may result in increased *p*-cresol production. This is consistent with the notion that the production of butyrate and *p*-cresol are inversely regulated.

*C*. *difficile* and other opportunistic gut bacteria have developed metabolic strategies that differ in response to environmental signals, one such strategy is the production of short chain fatty acids [38]. Studies using a simplified gnotobiotic mouse model, have shown that succinate produced by *B*. *thetaiotaomicron* is used by *C*. *difficile* to produce butyrate, boosting *C*. *difficile* titres [38]. However, in our experimental conditions (nutrient rich conditions) succinate was detected at very low levels by ^1^H NMR spectroscopy. Butyrate has anti-inflammatory properties [49] and is produced by a diverse array of bacterial phyla [50]. Yet, butyrate stimulates *C*. *difficile* toxin production in the absence of rapidly metabolised carbohydrates (*e*.*g*. glucose) [48]. The production of butyrate from acetyl-CoA or succinate by *C*. *difficile* is suppressed by the transcriptional regulators CcpA, CodY and Rex [51], however, if proline is limited then alternative pathways for NAD^+^ regeneration are used including glycine reductase, alcohol dehydrogenase and butyrate production from acetyl-CoA and succinate are induced [51]. We observed lower butyrate and isobutyrate concentrations in the 630Δ*erm* cultures (mono- and co-cultures), which implies that *C*. *difficile* is unable to synthesise butyrate under these conditions. However, we observed high butyrate and isobutyrate concentrations in co-culture with the *p*-cresol mutant and complement, where the competitor is more abundant, suggesting that the competitors are responsible for the increase in butyrate in co-culture.

Inter-*C*. *difficile* strain variation in the metabolic profiles included altered abundance of alanine, isobutyrate, *p*-cresol and *p-*HPA. We observed an increase in the production of *p*-cresol in monocultures and co-cultures containing the *C*. *difficile* 630Δ*erm* and an absence of *p*-cresol in all cultures with the *hpdC*::CT mutant, consistent with the inability of the mutant to synthesise *p-*cresol (Fig 5B). The complement produced an intermediate amount of *p-*cresol (Figs 5B and S3) and therefore clustered between the wild type and mutant in the PCA plot, suggesting that *p*-cresol and *p*-HPA were the main metabolites driving separation between *C*. *difficile* strains. This observation was corroborated when *p*-cresol production was increased in co-culture by increasing the supply of *p*-HPA, which resulted in a decrease in the competitor relative to wild-type *C*. *difficile*.

In this study, we present evidence that *p-*cresol production by *C*. *difficile* prevents outgrowth of discrete taxa of bacteria *in vitro* and that *p*-cresol production may modulate composition of the mouse microbiota. Our data showed clear differences in the composition of the microbiota of mice infected with the *p*-cresol mutant compared with the 630Δ*erm* strain pre- and post-relapse in our infection model (Fig 7). Recovery of the microbial community to its pre-dysbiotic state is often a slow process and, consequently, susceptibility to *C*. *difficile* colonisation can be increased for weeks and even months following cessation of antibiotics[52, 53]. Both the 630Δ*erm* and the *hpdC*::CT mutant successfully colonised mice at the initial infection stage. However, we observed increased microbial diversity in *hpdC*::CT infected mice, at day 7 post-infection. This diversity was largely driven by OTUs that each constituted <0.1% of the total microbiota. Despite relatively low abundance of these OTUs, they could have important consequences for the microbial ecosystem. These Families include Corynebacteriaceae, Propionibacteriaceae (both Actinobacteria), Bradyrhizobiaceae, Burkholderiaceae, Comamonadaceae, Oxalobacteraecea, Rhodocyclaceae, Bdellovibrionaceae and Enterobacteriaceae (all Proteobacteria). Consistent with the notion that *p*-cresol prevents outgrowth of Proteobacteria, the majority of these Families were members of the Proteobacteria Phylum. Vancomycin treatment reduced microbial diversity, altered the metabolic content of the stool samples, and resulted in a microbiome that was susceptible to relapse with *C*. *difficile*. The remaining microbial community was dominated by Lactobacillaceae (consistent with previous publications [24]), which was insufficient to restore colonisation resistance following vancomycin withdrawal. Upon cessation of vancomycin treatment, we observed an expansion of microbial diversity (D2R and D4R). There were clear differences in microbiota composition at D4R between animals infected with the *p*-cresol mutant and 630Δ*erm C*. *difficile*. The second most abundant class present in the microbiome of *hpdC* mutant-infected mice was Gammaproteobacteria (26.2%). In contrast, the second most abundant class in animals infected with 630Δ*erm C*. *difficile* was the Erysipelotrichia (16.5%). Other studies have shown that without FMT, dysbiosis is maintained in mice with two main OTUs, *Lactobacillus* and *Turicibacter* (Erysipelotrichia order)[24, 35], which we observed in the 630Δ*erm* infected mice, but not mice infected with the *p*-cresol mutant. We have shown *in vitro* that Gammaproteobacteria, including *K*. *oxytoca*, *E*. *coli* and *P*. *mirabilis* are more sensitive to *p*-cresol than *C*. *difficile*, while Gram-positive bacteria from the Lactobacillales family are more resistant to *p*-cresol. This is particularly pertinent as the majority of the microbiota post-vancomycin treatment was comprised almost exclusively of *Lactobacillus* and an increased expansion of the Gammaproteobacteria was only seen in *p*-cresol mutant-infected mice. The faecal metabolic profiles from all animals post-vancomycin treatment (D0R) were clearly distinct from those collected post-infection (D2, D4 and D7; Fig 7). However, the metabolic profiles of mice infected with the 630Δ*erm* strain 4 days after withdrawal of vancomycin were more variable than those infected with the mutant strain. The 630Δ*erm* infected mice also had a metabolic signature more similar in composition to those samples collected post-infection (D2, D4 and D7). This suggests that the biomolecular perturbations following re-establishment of infection with 630Δ*erm* were more closely related to those observed with the initial infection. In contrast, the *hpdC*::CT-infected mice had profiles more closely related to the uninfected mice.

To complement the *in vitro* co-culture assay and mouse model of CDI we assessed the effect of exogenous *p*-cresol on the human microbiota using *ex vivo* healthy human faecal samples. We observed a reduction in the number of viable total anaerobes, facultative anaerobes and lactose fermenting enterobacteriacea (LFE). The LFE are comprised of the Gammaproteobacteria *E*. *coli*, *Klebsiella spp*, *Enterobacteria spp*, *Citrobacter spp* and *Serratia spp*. This observation corroborates previous findings that demonstrate a significantly reduced viability of Gram-negative bacteria by *in vitro* growth kinetic analysis, competitive co-culture and in a mouse model of CDI. In contrast, the Gram-positive bacteria isolated from the *ex vivo* healthy human faecal samples (*Bifidobacteriaceae*, *Lactobacillales* and *Enterococcaceae*) were consistently less sensitive to *p*-cresol in the assays we performed.

In this study, we have shown that *C*. *difficile* displays a greater degree of tolerance to *p-*cresol when compared to a selection of other common intestinal bacterial species. Our data suggest a clear distinction between the fundamental properties of the organisms susceptible to the *p-*cresol, whereby Gram-positive species displayed greater tolerance than Gram-negative species. We demonstrate that *p*-cresol affects the integrity of surface barriers resulting in a concentration-dependant leakage of small molecules such as phosphate. Similar effects have been observed with *m*-cresol and chloro-cresol on bacterial cell membranes[29]. *p*-cresol was recently shown to inhibit proliferation of colonic epithelial cells and induce necrotic leakage of protons through the inner mitochondrial membrane[54]. *Pseudomonas putida* strain P8, which has the capacity to degrade *p-*cresol, modifies its fatty acid composition by increasing the abundance of 9-*trans* hexadeconoic acid and decreasing the abundance of 9-*cis*hexadeconoic acid when grown in the presence of sub*-*lethal concentrations of phenol[30, 55, 56]. Previous work has demonstrated that sublethal concentrations of phenolics, including *p*-cresol, resulted in an increase in the degree of saturation of cell membrane lipids, which is thought to counteract the increase in membrane fluidity [30, 57].

In conclusion, we demonstrate that the production of *p*-cresol by *C*. *difficile* alters the composition and recovery of diversity in the intestinal microbiota. A *p*-cresol deficient mutant has a reduced ability to compete with other intestinal microbiota species *in vitro*. We have shown that the effect of *p*-cresol is more detrimental to the growth of Gram-negative bacteria, differentially inhibiting proliferation of various bacterial Phyla. Exposure to *p-*cresol resulted in release of cellular phosphate, suggesting that it disrupts cell envelope integrity. This study provides evidence that *p*-cresol production by *C*. *difficile* provides it with a competitive survival advantage over other intestinal bacterial species.

## Materials and methods

### Bacterial strains and culture

*C*. *difficile* strains 630Δ*erm*[58] and *hpdC*::CT[22] have been previously described. The intestinal microbiota species used in the study were obtained from Mark Wilcox and Simon Baines at the University of Leeds isolated from a gut soup model of CDI (S1 Table). All bacteria were cultured in pre-reduced Brain Heart Infusion (BHI) (Oxoid), supplemented with 0.5% (w/v) yeast extract (BHIS) and 0.05% (w/v) L-cysteine (Sigma), at 37°C and under anaerobic conditions. For growth rate analysis, our collection of gut bacteria was grown in 100 ml tissue culture flasks with shaking at 50 rpm, 37°C and under anaerobic conditions. Pre-reduced growth media was supplemented with 0.1%, 0.05% and 0.01% (v/v) *p-*cresol as indicated. OD_595_ was determined every hour for 8 hours with a final reading at 24 hours, growth curves were performed in triplicate. A *p*-cresol complement strain (*hpdC*::CT::p*hpdCA*) was made, using an inducible plasmid based system derived from pRPF185[59] (S2 Table). The *hpdCA* genes were PCR amplified and cloned downstream of a tetracycline inducible promoter (p_tet_) in pRPF185 [59] to produce the plasmid p*hpdCA*. This was then conjugated into the *hpdC*::CT mutant to create a complement[60]. This was performed alongside an empty plasmid pLDempty, which was derived from pRPF185[59], to contain the p_tet_ promoter, but without a gene (S2 Table). This was transferred into *C*. *difficile* using competent *E*.*coli* CA434[60] into both 630Δ*erm* and *hpdC*::CT mutant as controls. Linear regression analysis was performed using Stata15; data was transformed using Log_10_ to approximate a normal distribution. The data was mined to determine if there was a significant difference in; a) strains, the growth of all bacteria strain compared to the reference strain *C*. *difficile* strain 630, b) *p*-cresol concentration compared to the BHIS untreated control, c) the Gram-negative bacteria compared to the Gram-positive bacteria. The COV indicates whether the growth is higher (positive number) or lower (negative number) than the reference and the *p*-value indicates the probability, a minimum cut off of *p*<0.05 was used throughout for significance (S3 Table).

#### Minimum inhibitory concentrations (MIC) assays

All experiments were performed in pre-reduced media. Primary *C*. *difficile* strains were grown in BHIS broth until OD_595_ 0.3 on a shaking platform at 50 rpm, then were diluted 1/100 into 24-well plates containing pre-reduced BHIS media supplemented with vancomycin or cefoperazone at a range of 1–256 μg/ml. These plates were incubated on a shaking platform at 50 rpm for 24 hours under anaerobic conditions at 37°C before OD_595_ was used to measure growth compared to an un-inoculated blank control. The MIC was taken as the lowest concentration to completely inhibit visible growth. The MICs were calculated as the mean of the three independent biological and technical replicates. There is no significant difference in the MICs between the *C*. *difficile* strains (630Δerm, *hpdC*::CT, *hpdC*::CT_p*hpdCA*) (S6 Table).

#### Growth rate analysis

All experiments were performed in pre-reduced media. Growth rates of *C*. *difficile* strains (630Δ*erm*, *hpdC*::CT and *hpdC*::CT::p*hpdCA*) and intestinal species (*E*. *coli*, *K*. *oxytoca*, *E*. *faecium* and *L*. *fermentum*), were grown for 8 hours in BHIS media to compare growth rates, by measuring OD_595_. CFUs were calculated at OD 0.5 for each of the strains tested (S4 Table). The effect of anhydrotetracycline (ATC) was assessed by growing overnight primary cultures of *C*. *difficile* strains (630Δ*erm*, *hpdC*::CT and *hpdC*::CT::p*hpdCA*) in 10 ml BHIS supplemented with 0, 100 and 250ng/ml ATC. OD_595_ was measured and each strain diluted to an OD_595_ 0.5, and used to inoculate secondary cultures at OD_595_ 0.05 into pre-reduced BHIS, supplemented with ATC as outlined above. The OD_595_ was determined every hour for 8 hours with a final reading at 24 hours, growth curves were performed in triplicate.

#### Co-culture with extrinsic *p*-cresol

Under anaerobic conditions, an overnight culture of *C*. *difficile* and a gut competitor were normalised to a starting OD_595_ of 0.5 and were used to inoculate 1/10 into 15 ml of BHI + 0.05% (w/v) L-cysteine supplemented with 0.05% (v/v) *p-*cresol, which was grown with shaking (50 rpm) for 24 hours. The total number of colony forming units (CFU) was determined by plating serial dilutions on non-selective BHI + 0.05% (w/v) L-cysteine. The total number of *C*. *difficile* was determined by plating serial dilutions on BHIS + 0.05% (w/v) L-cysteine and cycloserine/cefoxitin supplement (BHIS CC) (Sigma), which could, in turn, be used to determine the total number of CFU for the competitor. Serial dilutions were plated in triplicate and an average of the three technical replicates was used to determine total CFU. Each experiment was performed in triplicate. Linear regression analysis was performed in Stata15 on the log_10_ of the CFU as outlined above.

#### Competitive co-culture with intrinsic *p*-cresol

Under anaerobic conditions, individual overnight cultures of *C*. *difficile* and an intestinal competitor were grown in 10 ml of BHI + 0.05% (w/v) L-cysteine supplemented with 100 ng/ml or 250 ng/ml ATC (to induce expression of *hpdCA* expressed *in trans* in the complement strain). Individual monocultures were normalised to a starting optical density (OD_600_) of 0.5, and were inoculated 1/10 into BHIS broth supplemented with 0.05% L-cysteine (w/v) and either 0.1, 0.2 or 0.3% *p-*HPA (w/v) (6.5 mM, 13.1 mM and 19.7 mM, respectively), these monocultures were grown until *C*. *difficile* reached an OD 0.5–0.6 (~7 hours). The competitor was back diluted to an OD_595_ 0.5 and inoculated 1:10 into the *C*. *difficile* culture, to create a competitive co-culture. These co-cultures were grown anaerobically, shaking (50 rpm) for 24 hours and were plated onto both BHIS non-selective plates and BHIS CC plates. CFU counts of both *C*. *difficile* and the competitor were determined and processed as outlined above. Each experiment was performed in triplicate and Linear regression analysis was performed in Stata15 on the log_10_ of the CFU as outlined above, statistically significant differences were observed *p*<0.05.

### Metabolic profiling of supernatants

Culture supernatant from mono-culture and co-culture experiments in media supplemented with 0.1 and 0.2% (v/v) *p*-HPA were filter sterilised. Samples were diluted into 400 μL of phosphate buffer (pH 7.4, 100% D_2_O, 3 mM of NaN_3_, 1 mM of 3-(trimethyl -silyl)-[2,2,3,3-^2^H4]-propionic acid (TSP) for the chemical shift reference at δ0.0) according a 1:2 ratio. Samples were transferred to 5 -mm tubes for ^1^H nuclear magnetic resonance (NMR) spectroscopic analysis, which was performed on a Bruker 600 MHz spectrometer (Bruker Biospin, Karlsruhe, Germany) at 300K (26.85°). The parameters of the acquisition were as previously reported for urine[61]. Each spectrum was acquired with 4 dummy scans followed by 32 scans. Spectra were automatically phased, baseline corrected and calibrated to the internal standard (TSP) using Topspin (Bruker Biospin, Karlsruhe, Germany). The processed spectral data was imported into Matlab (version R2014a, The Mathworks Inc.). The region δ4.84–4.76 was removed to eliminate the residual water signal. Principal Components Analysis (PCA) was performed using pareto scaling, due to the significant intensity of the acetate signal (δ 1.92, single. Based on the PCA loadings, spectral peaks contributing to the principal components were integrated using an in-house script. These metabolite peak integrals were used to construct a clustergram in Matlab using the clustergram script.

### Sporulation assays

Primary cultures were inoculated from a single colony of *C*. *difficile* strains (630Δ*erm*, hpdC::CT, *hpdC*::Ctp*hpdCA*) into pre-reduced BHIS broth and grown to an OD_595_ 0.3 on a shaking platform at 50 rpm. These were inoculated 1/100 into pre-equilibrated BHIS broth which was incubated statically for 72 h under anaerobic conditions at 37°C. Total counts (vegetative cells and spores) and spore counts were then determined using CFU assays in 1X PBS (1/10 dilutions from 0 to -5). All dilutions were plated onto BHIS plates supplemented with 1% taurocholate. The spore counts were performed by heat inactivation of vegetative cells at 65°C for 20 minutes, these were then serially diluted and CFU counts determined on BHIS taurocholate plates. All experiments were performed with duplicate technical replicates and triplicate biological replicates. All data was analyzed in Excel, plotted in GraphPad Prism 7 and statistical analysis was performed in Stata15 using regression analysis p<0.05 were considered significantly different (S5 Fig).

### Mouse relapse model of CDI

Female C57BL/6 mice (Charles River; 7–9 weeks old) were kept in independently ventilated cages under sterile conditions. As outlined by Theriot *et al*.[62], mice were treated with cefoperazone in the drinking water (50 mg/litre) for 10 days to disrupt their normal microflora, rested for two days, before they were infected with 10^4^ *C*. *difficile* spores by oral gavage. After 28 days, vancomycin was added to the drinking water for 7 consecutive days (400 mg/litre) to induce relapse of CDI. Fresh faecal samples from individually infected mice were collected throughout the time course to be utilised for determining the *C*. *difficile* load, 16S rRNA sequencing of the microflora and metabolite profiling by ^1^H spectroscopy. Stool samples were plated onto *C*. *difficile* selective plates to determine the bacterial load (CFU/g). Statistical analysis was performed using a one tailed Mann Whitney U test, *p*<0.05 were considered significantly different.

### Ethics statement

All animal procedures were performed at Royal Holloway in accordance with the Home Office project license PPL 70/8276, that enables work to be conducted under the UK “Animal (Scientific Procedures) Act 1986”. This work was approved by the Royal Holloway, University of London Ethics Committee.

Healthy human donor faecal samples were collected and processed using different healthy donors who had not received antibiotic treatment in the preceding 3 months in accordance with the University of Hertfordshire Ethics committee guidelines and approval (UH Ethics Approval Number: aLMS/SF/UH/00103). All donors provided informed written consent.

### Microbiota analysis

DNA was extracted from faecal samples using a combined method based on phenol:chloroform:isoamyl alcohol extraction, ethanol precipitation and FastDNA SPIN kit for soil (MPBiomedicals). Briefly, an equal mass of faecal material was suspended in 50 mM Tris-HCl pH7.5, 10 mM EDTA, homogenised and bacterial cells were lysed using a FastPrep-24 *Classic* Instrument (MPBiomedicals). Nucleic acid was extracted using a standard phenol:chloroform:isoamyl alcohol procedure, followed by ethanol precipitation and was suspended in nuclease free dH_2_O. Faecal DNA was subsequently purified using the DNA binding matrix from the FastDNA SPIN kit for soil with minor modifications of the manufacturer’s instructions. Briefly, DNA samples were added to sodium phosphate buffer, MT buffer and protein precipitation solution supplied in the kit and this was added directly to the binding matrix. DNA was subsequently purified according to the manufacturer’s instructions and eluted in DNase-free water. Library preparations for the MiSeq were performed as outlined in Rosser *et al*[63]. Briefly, an amplification step was used to add Illumina compatible adaptors, with a unique 12 bp individual barcodes for each sample, with an extra pad and linker sequence. The V5-7 regions of the 16S rRNA genes were then amplified using 785F: 5ʹ-GGATTAGATACCCBRGTAGTC-3ʹ, 1175R: 5 ʹ-ACGTCRTCCCCDCCTTCCTC-3ʹ primers, where the reverse primer (1175R) contained the individual error-corrected barcode: 25 μl reactions were comprised of 1x Molzym PCR buffer, 0.025 μM Moltaq (Molzym), 200 μM dNTPs (Bioline), 0.4 μM forward and reverse primer, 2 μl DNA and nuclease free water (Bioline)[63]. Cycling parameters for each reaction were 94°C x 3 min, then 30 cycles of 94°C x 30 s, 60°C x 40 s, 72°C x 90 s and final extension at 72°C for 10 min. Samples were purified and normalised using a SeqPrep normalisation plate kit (Invitrogen), and quantified using a Qubit2.0 (Life technologies), and further purified using 0.6 X Agencourt AMPure Beads (Beckman Coulter), a selection of samples were run on an Agilent high sensitivity DNA chip (Agilent Technologies), samples were quantified again using a Qubit 2.0 (Life Technologies), and were pooled in equimolar solution, then diluted to a 2 nM library, with 10% PhiX control and loaded into the MiSeq run cartridge in accordance with the manufacturer’s instructions (Illumina). The MiSeq runs produced 250 bp paired end reads, with a 12 bp individual index for each sample. The sequence reads generated were de-multiplexed and quality filtered using QIIME (version 1.9.1 [64]) following the standard pipeline to assign Illumina reads to operational taxonomic units (OTUs) using the Greengenes database [65]). Associated summaries and diversity analyses were also performed in QIIME. Subsequent analyses were performed in R [66] and visualised with ggplot2 [67]. We selected families to include in our 16S plots (Fig 7A) if they had mean proportion of greater than 1% in any of the 12 day/type phenotype combinations. Box plots (Fig 7B) and PCA (Fig 7C) were calculated from the full 16S rRNA sequence dataset at the family level. ANOSIM analysis was used to identify variation in species abundance and composition between strains 630Δ*erm* and *hpdC*::CT, as well as between time points D7, D0R, D2R and D4R. Significant differences were indicated with a circle *p*<0.001.

### Metabolic profiling of mouse faecal samples

Faecal samples were defrosted and mixed with 400 μL of phosphate buffer (pH 7.4, 100% D_2_O, 3 mM of NaN_3_, 1 mM of 3-(trimethyl-silyl)-[2,2,3,3-^2^H4]-propionic acid (TSP) for the chemical shift reference at δ0.0) and Zirconium beads (0.45 g ±0.1). The samples were vortexed and then homogenised with a FastPrep-24 *Classic* Instrument (MP BIOMEDICALS) (30 sec per cycle, speed 6.0, 2 cycles). After a centrifugation (13,000 x*g*, 15 min), 180 μL of the supernatants were collected and transferred in 3-mm tubes for ^1^H nuclear magnetic resonance (NMR) spectroscopic analysis, which was performed on a Bruker 600 MHz spectrometer (Bruker Biospin, Karlsruhe, Germany) at 300K (26.85°). The parameters of the acquisition were as previously reported for urine[61]. 4 dummy scans followed per 64 scans were acquired for each spectrum which were then imported into Matlab (version R2014a, The Mathworks Inc.). The region δ4.82–4.76 was removed to eliminate residual water signal. All spectra were normalised according probabilistic quotient method and automatically aligned. Principal Components Analysis (PCA) was performed with mean-centring and Pareto scaling.

### HPLC

Frozen culture supernatants were defrosted on ice and were mixed in a 1:1 ratio with methanol: water, transferred to HPLC tubes and processed immediately by HPLC. Mouse faecal samples were defrosted, and added to a 2 ml screw cap tube containing 2 mm beads. These were weighed before and after addition of the faecal sample. To these, 400 μl 1:1 methanol:water was added to the pellet, then ribolysed twice using a FastPrep-24 *Classic* Instrument at speed 6.0 m/s for 30 sec. Tubes were transferred to ice and centrifuged at 14000 x*g* for 20 minutes. 250 μl of the supernatant was transferred to a clean sterile HPLC tube and were transferred immediately for HPLC. Each experiment was performed in triplicate. All HPLC equipment, software, solvents, columns and vials were from Thermo Fisher Scientific, UK. Separations were performed utilising an Acclaim 120, C18, 5 μm Analytical (4.6 x 150 mm) and the mobile phase consisting of ammonium formate (10 mM, pH 2.7) and menthol (v/v; 40:60) at a flow rate of 2 ml/min. *p-*HPA and *p*-cresol were detected by the photo-diode array detector (UV-PDA; DAD 3000) set at 280 nm. Peak identity was confirmed by measuring the retention time, spiking the sample with commercially available *p-*HPA and *p*-cresol and determination of absorbance spectra using the UV-PDA. A calibration curve of each compound was generated by Chromeleon (Dionex software) using known amounts of the reference standards (0–100 mg/ml) in methanol/water (v/v; 1:1) injected onto the column to determine the amount in the samples. The lower limit of detection was determined for *p-*HPA to be 0.03 mg/ml, for *p*-cresol to be 0.02 mg/ml. The concentration in mM was determined in Excel, using the molecular weight of the compounds and the quantity in mg/ml. The data was analysed in GraphPad Prism7 and statistical analysis was performed in Stata15 using linear regression analysis.

### Cultivatable human bacteria following *p*-cresol stress

Healthy human donor faecal samples were collected and processed using three different healthy donors who had not received antibiotic treatment in the preceding 3 months. Faecal samples (5 g) were emulsified in sterile pre-reduced PBS (50 ml) and faecal material was coarse filtered by passing the 10% emulsion through sterile muslin cloth to remove larger particle matter and leave a bacterial suspension. Faecal emulsions were incubated for 1 hour and 30 minutes in 1X PBS, or PBS containing 0.1% (v/v) *p*-cresol or 0.3% (v/v) *p*-cresol. Samples were then sedimented by centrifugation at 14000 x *g* for 5 minutes and the supernatant were removed. Pellets were resuspended in 1 ml 1X PBS and viable counts (CFU/ml) were performed on differentially selective agar, both anaerobically and aerobically. Each experiment was performed in triplicate. Serial 10-fold dilutions of re-suspended faecal emulsions in sterile pre-reduced peptone water were inoculated onto: fastidious anaerobe agar (total anaerobes), nutrient agar (total facultative anaerobes), kanamycin aesculin azide agar (Enterococci), LAMVAB agar (Lactobacilli), Beeren’s agar (Bifidobacteria), MacConkey agar number 3 (lactose-fermenting Enterobacteriaceae), *Bacteroides* bile aesculin agar (*B*. *fragilis* group), and total viable counts were determined in triplicate, and normalised to the starting CFU.

### Phosphate release

Release of cellular phosphate was investigated using a Colorimetric Phosphate Assay Kit (Abcam). The assay involved treating samples with ammonium molybdate and malachite green which forms a chromogenic complex with phosphate ions which can be detected at a wavelength of 650 nm. An overnight culture of each bacterial strain was sedimented by centrifugation and re-suspended in Tris-buffered saline (TBS, 50 mM Tris-HCl pH7.5, 150 mM NaCl) and subsequently washed two further times to remove traces of the growth medium. OD_595_ was determined and cell suspensions were normalised to an OD_595_ of 1.0. Five hundred microliter aliquots of cell suspension were sedimented by centrifugation and re-suspended in either TBS alone or TBS + *p*-cresol. Cell suspensions were incubated for the indicated time, cells were sedimented and 30 μl of supernatant was removed and added to 170 μl H_2_O and 30 μl ammonium molybdate and malachite green reagent. Absorbance was read at 650 nm in a 96-well microtitre plate reader. Phosphate release was determined by normalising the optical density from cell suspensions incubated with *p-*cresol against cell suspensions that were incubated with TBS alone. The assay was performed under anaerobic conditions except for spectrophotometry and sedimentation steps, for which tubes and flasks were sealed with parafilm to prevent oxygen infiltration. The maximum intracellular phosphate pool was determined by boiling a 500 μl cell suspension (OD_595_ 1.0) for 15 minutes. All assays were performed in triplicate.

## Supporting information

S1 Fig Differential growth rates in BHIS. Growth curves were performed in BHIS media under anaerobic conditions to determine the differential growth rate between *C*. *difficile* strains 630Δ*erm*, *hpdC*::CT and the complemented mutant (*hpdC*::CT::p*hpdC-A* and the intestinal microbiota species a) *E*. *coli* and *K*. *oxytoca*, and b) *E*. *faecium* and *L*. *fermentum*. The time, in minutes, that each strain reached OD_595_ 0.5 is indicated by the purple hash line. C) The growth rate of *C*. *difficile* strains (630Δ*erm*, *hpdC*::CT and complement *hpdC*::CT::p*hpdC-A*) was assessed in BHIS supplemented with a range of anhydrotetracycline concentrations (0, 100 and 250 ng/ml). All experiments were performed in triplicate. Error bars are Standard Error of Mean (SEM).
(TIF)

S2 Fig Modulating *p*-cresol production and the effect on competitive co-culture. Relative fitness of *C*. *difficile* 630Δ*erm*, *hpdC*::CT and *hpdC*::CT complement in competitive co-culture for 24 hours with gut commensal species was performed in media supplemented with *p-*HPA (0.3% v/v). Expression of the *hpdCA in trans* from a plasmid-borne tetracycline-inducible promoter in the *hpdC*::CT background was evaluated by varying the concentration of anhydrotetracycline (100 and 250 ng/ml). The relative fitness of the *C*. *difficile* strains (wild-type, *p*-cresol mutant and complement) was compared in competition with a) *E*.*coli* and b) *K*. *oxytoca*. The relative proportion of each strain was expressed as a percentage of the total CFU count. Error bars are representative of three independent replicates. Regression analysis was used to determine significant differences in growth taking strain into consideration and marked ** *p*<0.01 and ***p<0.001. c&d) The concentration of *p*-cresol produced in the co-cultures quantified by HPLC. e&f) The concentration of *p*-HPA remaining in the media after the co-cultures was quantified by HPLC. Regression analysis was used to determine significant differences in *p*-cresol production and *p*-HPA utilisation compared to 630Δ*erm* *** *p*<0.001. Error bars are SEM.
(TIF)

S3 Fig Detection of *p*-cresol by ^1^H NMR. a) ^1^H NMR spectra doublet of *p*HPA (δ6.867ppm) and *p*-cresol (δ6.831ppm), with *C*. *difficile* strains highlighted in different colours 630Δ*erm* (black), *hpdC*::CT (red) and the complemented mutant *hpdC*::CT::p*hpdC-A* (blue). Each line represents an individual ^1^H NMR spectrum of the culture supernatants. The arrows on the right indicate the differences between the *p*-cresol levels produced from either 0.1% *p*HPA or 0.2% *p*HPA.
(TIF)

S4 Fig Comparison of the metabolite profiles in medium supplemented with 0.1% and 0.2% *p*HPA. ^1^H NMR spectroscopy was used to determine the metabolite profiles of co-culture samples in media containing 0.1% and 0.2% *p-*HPA. a) PCA demonstrating metabolic variation across the profiles in media containing 0.1% or 0.2% (v/v) *p-*HPA. b) Loading plots of the PCA model showing the metabolites driving the groupings along principal component 1 (PC1) and principal component 2 (PC2).
(TIF)

S5 Fig Sporulation assay of *C*. *difficile* strains. The sporulation frequency of strains 630Δ*erm*, *hpdC*::CT and the complemented mutant *hpdC*::CT::p*hpdC-A*, were determine in BHIS media grown for 72 hours in BHIS broth. a) total cell counts were enumerated on BHIS plates containing 0.1% taurocholate and spore counts were determined by heat inactivation of vegetative cells at 65°C for 20 minutes before enumerated on BHIS taurocholates plates. b) percentage sporulation was calculated as a proportion of the total cell counts for each strain respectively. Statistical analysis was performed in Stata15 using linear regression and *p*<0.05 was considered a statistically significant difference. n/s indicates no significant differences.
(TIF)

S6 Fig ANOSIM population analysis of the microbiota. ANOSIM was performed to identify differences in bacterial population between 630Δ*erm* and *hpdC*::CT infected animals across the experiment as well as at given time points, D7, D0R, D2R and D4R. Significant differences are indicated with a circle *p*<0.001.
(TIF)

S1 Table Gut intestinal microbes used in this study. (XLSX)

S2 Table Strains, plasmids and primers. (XLSX)

S3 Table Statistical analysis of growth curves in BHIS with exogenous *p*-cresol. The top table shows the output of a Linear Regression analysis to compare the growth of a particular strain of bacteria, at a given concentration of *p*-cresol against the reference strain, *C*. *difficile*. Grey shading indicates analyses where the *p*<0.05. The bottom table indicates the relative effect of *p*-cresol concentration on the growth of a particular strain, relative to the growth rate in BHIS as a reference. This analysis was performed in Stata15 using linear regression, *p*<0.05 are shaded grey. COV is the co-efficient of variance of the test from the reference.
(XLSX)

S4 Table Colony counts for different bacterial strains at OD 0.5. (XLSX)

S5 Table Colony counts for extrinsic *p*-cresol co-culture experiments. CFU/ml was determined each bacterial species grown in co-culture in the presence or absence of extrinsic *p*-cresol.
(XLSX)

S6 Table Minimum inhibitory concentrations of vancomycin and cefoperazone. (XLSX)

S7 Table Diversity of the microbiome at Day 7. A table demonstrating the relative abundance of families comprising the faecal microbiome at day 7 for the wild-type infected, *hpdC*::CT infected and naïve mice.
(XLSX)
