# Supplementary figures and images for: Para-cresol production by Clostridium difficile affects microbial diversity and membrane integrity of Gram-negative bacteria
Source: PLoS Pathog. 2018 Sep 12;14(9):e1007191. doi: 10.1371/journal.ppat.1007191 (PMC6135563; doi:10.1371/journal.ppat.1007191)

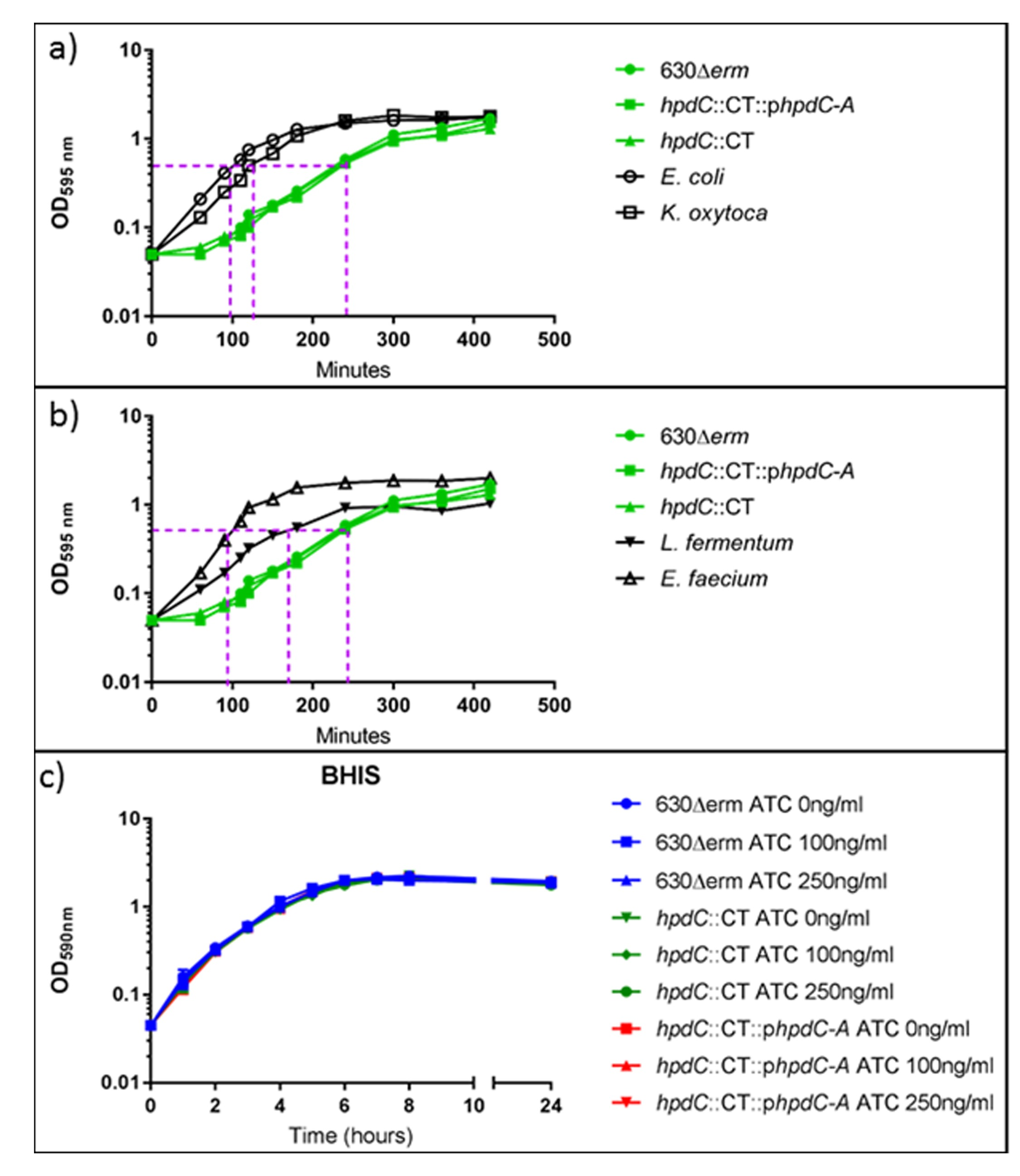

Supplement: S1 Fig — Growth curves were performed in BHIS media under anaerobic conditions to determine the differential growth rate between C. difficile strains 630Δerm, hpdC::CT and the complemented mutant (hpdC::CT::phpdC-A and the intestinal microbiota species a) E. coli and K. oxytoca, and b) E. faecium and L. fermentum. The time, in minutes, that each strain reached OD595 0.5 is indicated by the purple hash line. C) The growth rate of C. difficile strains (630Δerm, hpdC::CT and complement hpdC::CT::phpdC-A) was assessed in BHIS supplemented with a range of anhydrotetracycline concentrations (0, 100 and 250 ng/ml). All experiments were performed in triplicate. Error bars are Standard Error of Mean (SEM). (TIF) [file ppat.1007191.s001.tif]

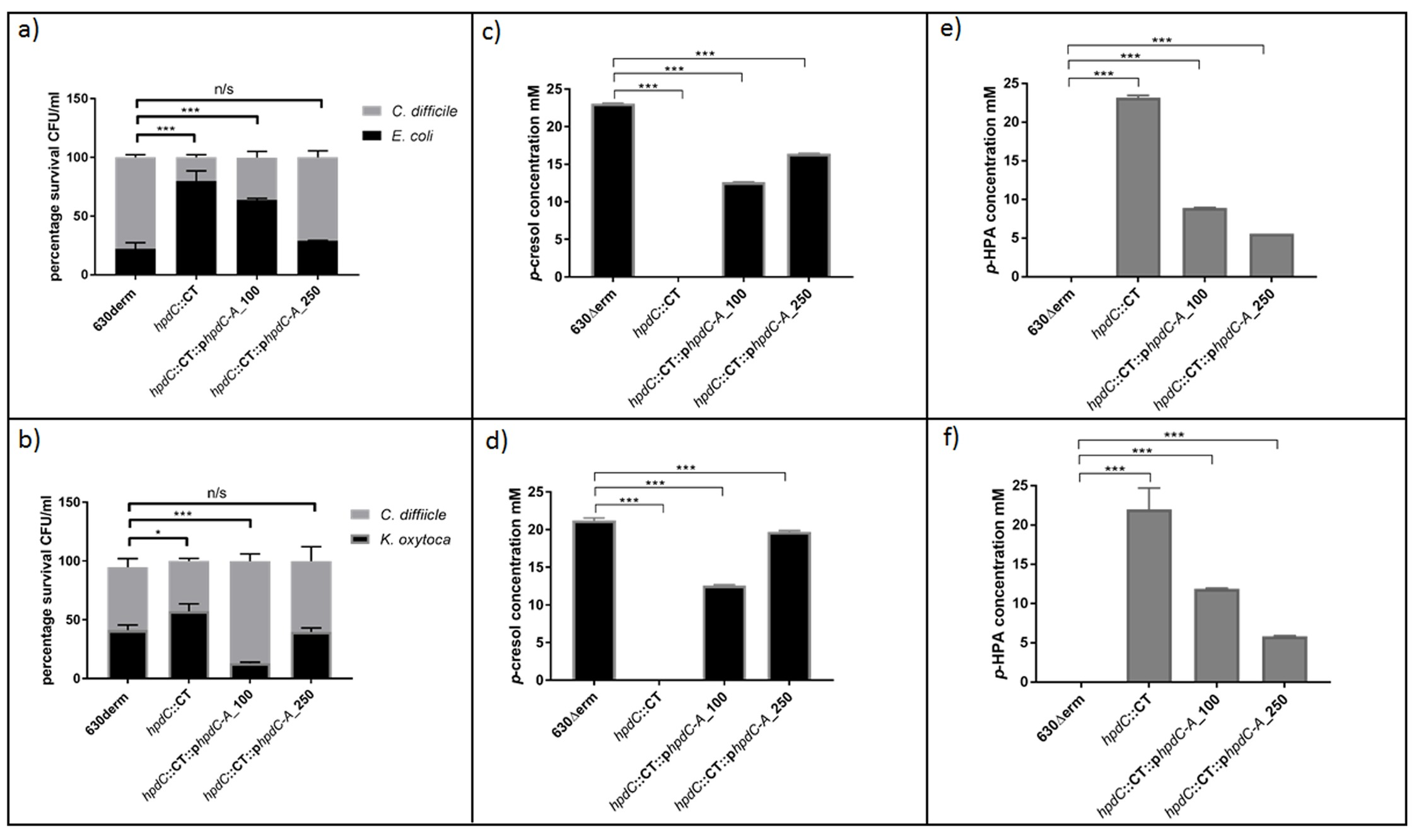

Supplement: S2 Fig — Relative fitness of C. difficile 630Δerm, hpdC::CT and hpdC::CT complement in competitive co-culture for 24 hours with gut commensal species was performed in media supplemented with p-HPA (0.3% v/v). Expression of the hpdCA in trans from a plasmid-borne tetracycline-inducible promoter in the hpdC::CT background was evaluated by varying the concentration of anhydrotetracycline (100 and 250 ng/ml). The relative fitness of the C. difficile strains (wild-type, p-cresol mutant and complement) was compared in competition with a) E.coli and b) K. oxytoca. The relative proportion of each strain was expressed as a percentage of the total CFU count. Error bars are representative of three independent replicates. Regression analysis was used to determine significant differences in growth taking strain into consideration and marked ** p<0.01 and ***p<0.001. c&d) The concentration of p-cresol produced in the co-cultures quantified by HPLC. e&f) The concentration of p-HPA remaining in the media after the co-cultures was quantified by HPLC. Regression analysis was used to determine significant differences in p-cresol production and p-HPA utilisation compared to 630Δerm *** p<0.001. Error bars are SEM. (TIF) [file ppat.1007191.s002.tif]

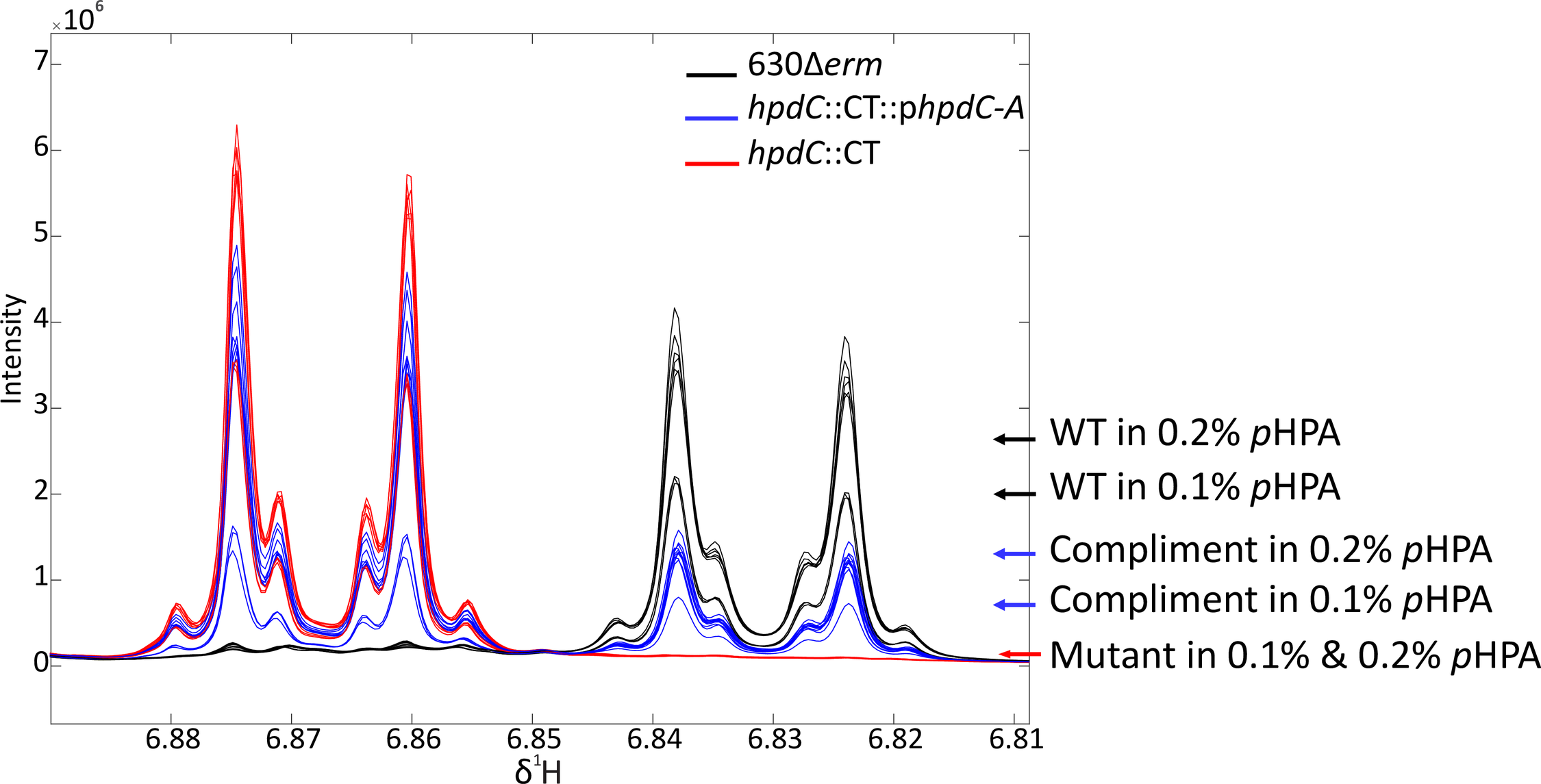

Supplement: S3 Fig — a) 1H NMR spectra doublet of pHPA (δ6.867ppm) and p-cresol (δ6.831ppm), with C. difficile strains highlighted in different colours 630Δerm (black), hpdC::CT (red) and the complemented mutant hpdC::CT::phpdC-A (blue). Each line represents an individual 1H NMR spectrum of the culture supernatants. The arrows on the right indicate the differences between the p-cresol levels produced from either 0.1% pHPA or 0.2% pHPA. (TIF) [file ppat.1007191.s003.tif]

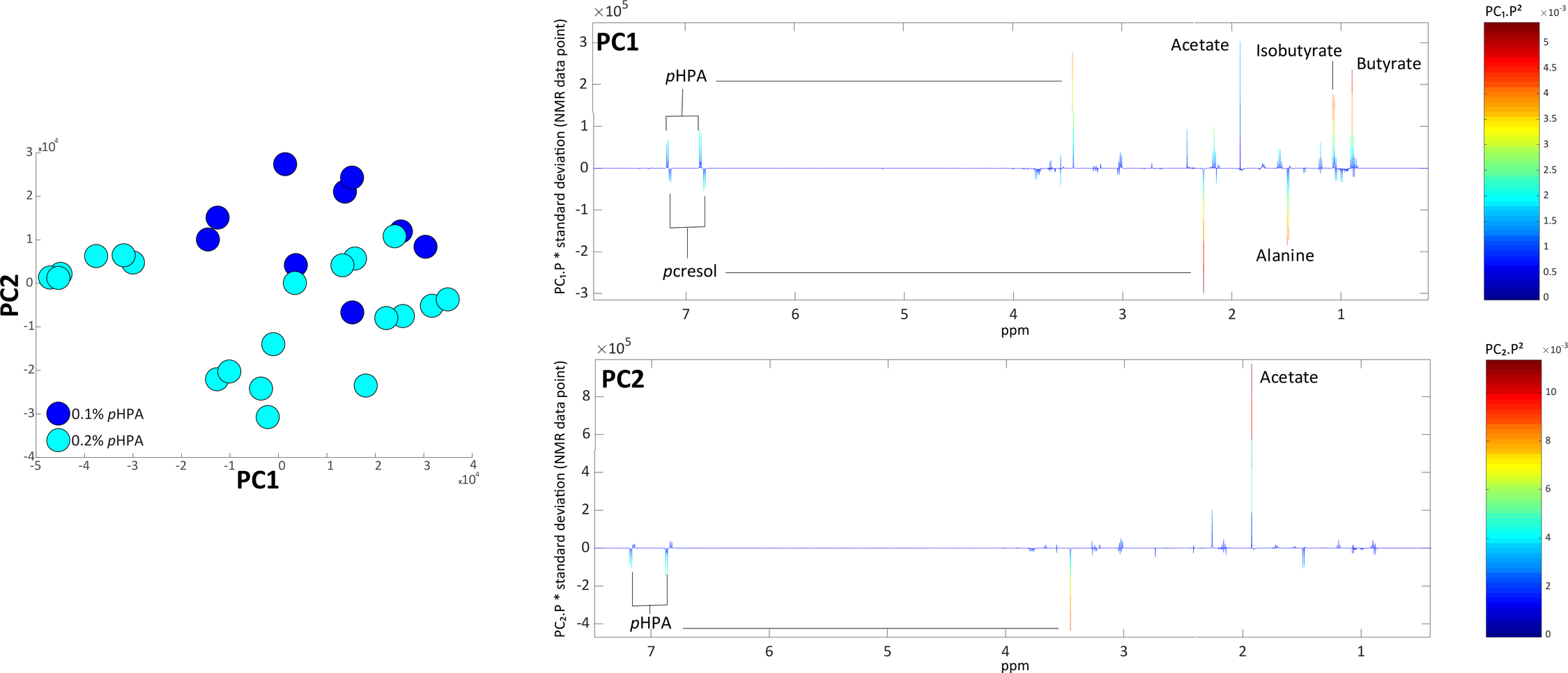

Supplement: S4 Fig — 1H NMR spectroscopy was used to determine the metabolite profiles of co-culture samples in media containing 0.1% and 0.2% p-HPA. a) PCA demonstrating metabolic variation across the profiles in media containing 0.1% or 0.2% (v/v) p-HPA. b) Loading plots of the PCA model showing the metabolites driving the groupings along principal component 1 (PC1) and principal component 2 (PC2). (TIF) [file ppat.1007191.s004.tif]

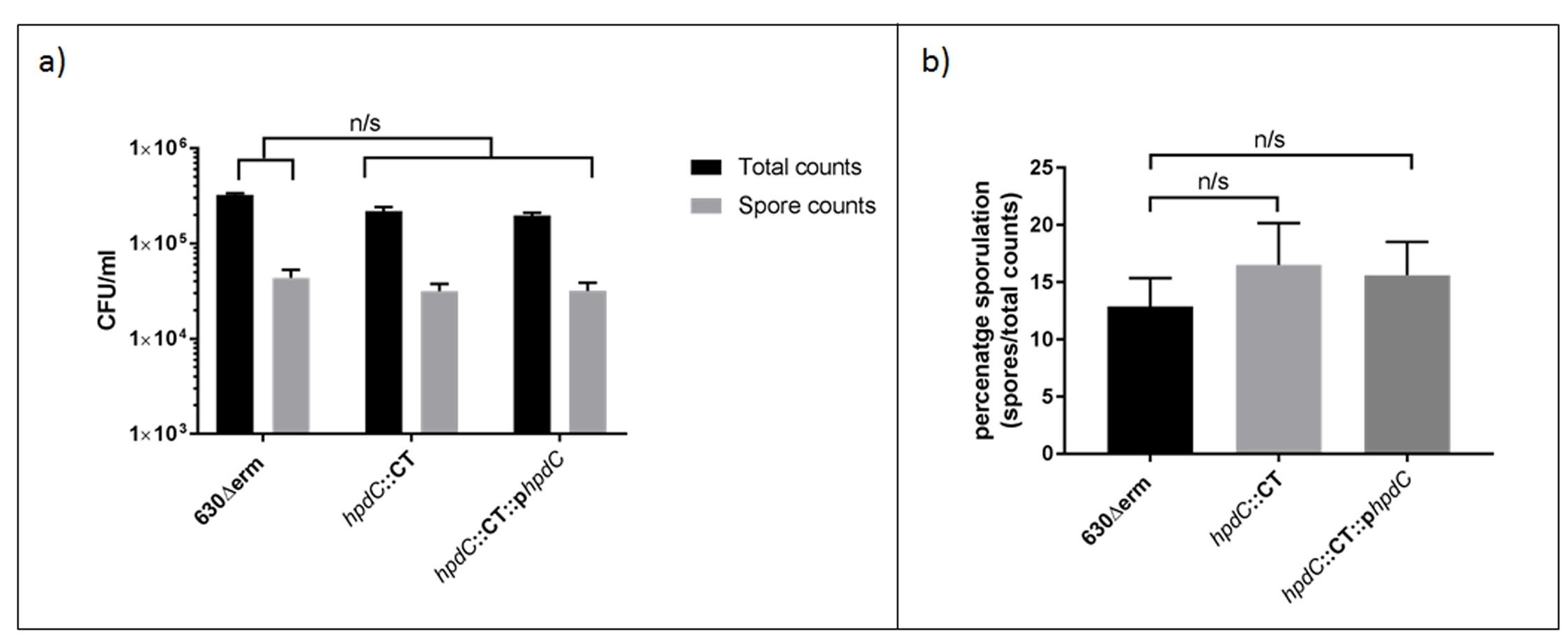

Supplement: S5 Fig — The sporulation frequency of strains 630Δerm, hpdC::CT and the complemented mutant hpdC::CT::phpdC-A, were determine in BHIS media grown for 72 hours in BHIS broth. a) total cell counts were enumerated on BHIS plates containing 0.1% taurocholate and spore counts were determined by heat inactivation of vegetative cells at 65°C for 20 minutes before enumerated on BHIS taurocholates plates. b) percentage sporulation was calculated as a proportion of the total cell counts for each strain respectively. Statistical analysis was performed in Stata15 using linear regression and p<0.05 was considered a statistically significant difference. n/s indicates no significant differences. (TIF) [file ppat.1007191.s005.tif]

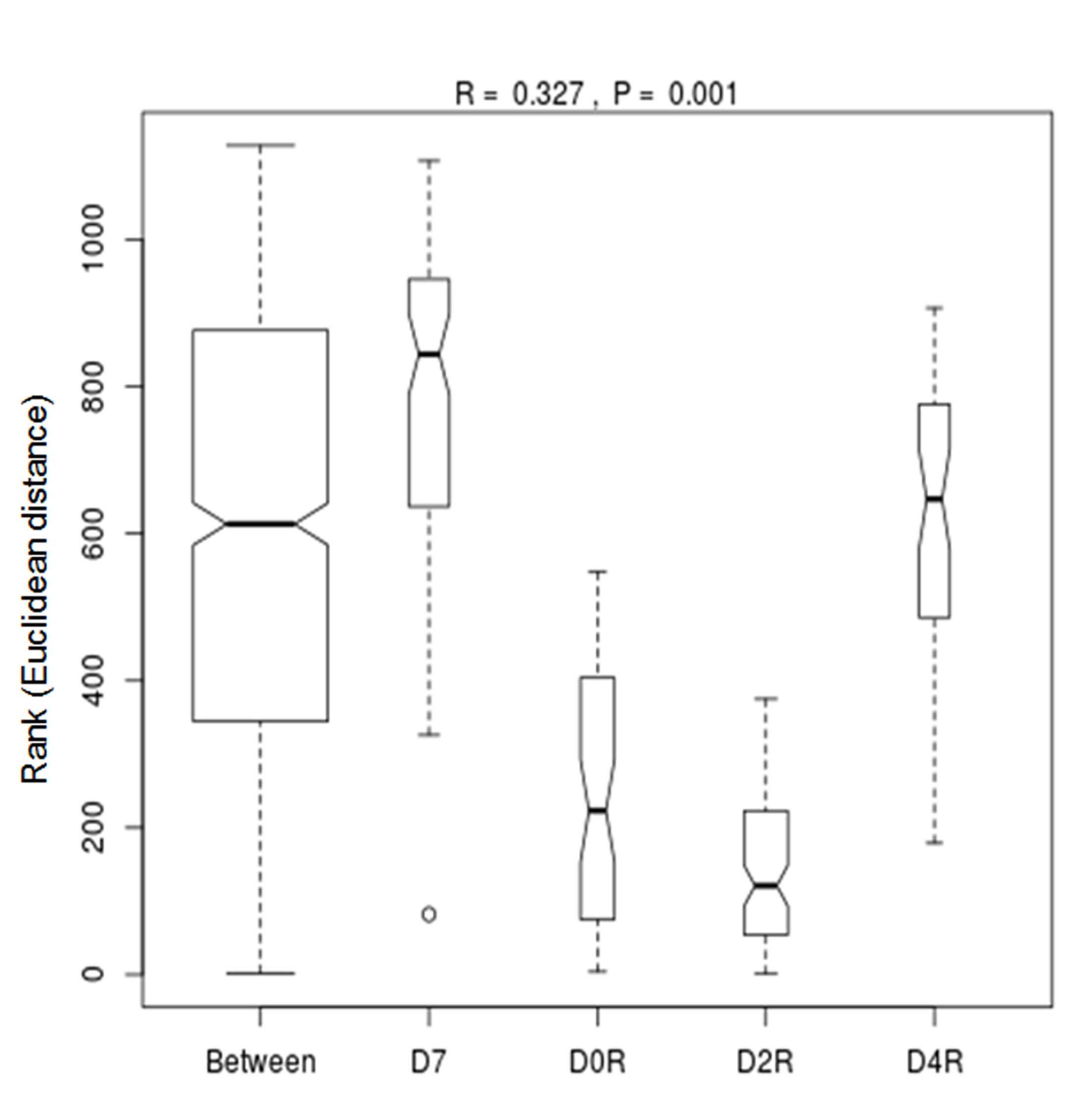

Supplement: S6 Fig — ANOSIM was performed to identify differences in bacterial population between 630Δerm and hpdC::CT infected animals across the experiment as well as at given time points, D7, D0R, D2R and D4R. Significant differences are indicated with a circle p<0.001. (TIF) [file ppat.1007191.s006.tif]
